# First brassinosteroid-based dwarf mutant discovered and characterized in grapevine

**DOI:** 10.1007/s00122-026-05225-6

**Published:** 2026-05-04

**Authors:** Yingzhen Yang, Jie Arro, Cheng Zou, Madeline Oravec, Bruce Reisch, Gan-Yuan Zhong

**Affiliations:** 1 Grape Genetics Research Unit, USDA-Agricultural Research Service, Geneva, NY 14456 USA; 2 Institute of Biotechnology, Cornell University, Ithaca, NY 14850 USA; 3 School of Integrative Plant Science, Cornell AgriTech, Cornell University, Geneva, NY 14456 USA; 4 Present Address: Sun World International, 28994 Gromer Ave, Wasco, CA 93280 USA; 5 Present Address: Biology Department, University of St. La Salle, Bacolod City 6100, Philippines

## Abstract

In this study, we investigated the genetic control of dwarfism in naturally occurring dwarf mutant lines of grapevines. Through trait–segregation and marker–trait association analyses, we identified a major locus on Chromosome 14 tightly associated with the dwarf trait. Subsequently, we conducted a bulked RNA-seq analysis, fine mapped the dwarf trait and identified *VviBR6OX1*, a cytochrome P450 enzyme involved in brassinosteroid synthesis, as a candidate gene for the observed dwarfism. RNA-seq sequence analyses revealed two in-frame deletions in the gene: a 12-bp deletion in exon 1 and a 9-bp deletion in exon 4. A survey of the two indels in *Vitis* germplasm suggested that the 9-bp deletion is most likely the cause of dwarfism in the mutant. We recreated similar dwarf grapevines by knocking out *VviBR6OX1* using CRISPR/Cas9 gene editing and confirmed *VviBR6OX1*’s role in controlling vine architecture. Additionally, we observed several vines with an extreme compact dwarf phenotype and determined that the compact dwarfing phenotype was a result of simultaneous editing of a second *BR6OX* gene, *VviBR6OX2*. The discovery of BR-related dwarfism in grapevine provides an important genetic avenue for developing desirable vine architecture for various breeding purposes.

**Supplementary Information:**

The online version contains supplementary material available at 10.1007/s00122-026-05225-6.

## Introduction

Dwarfing is a highly valued agronomic trait for dense planting, lodging resistance and yield improvement for many field crops. Likewise, it is also an important attribute in woody species for the control of plant architecture, size and productivity. There are various genetic factors controlling dwarfism. Gibberellins (GA) and brassinosteroids (BR) are two major types of plant hormones known as master regulators of plant development, particularly influencing plant height through their roles in cell expansion, elongation and orientation. Disruptions in either GA or BR biosynthesis or signaling pathways can lead to reduced plant growth and dwarfism (Tong et al. 2014). The first Green Revolution in the 1960s leveraged natural rice and wheat dwarf mutants, which were defective in GA biosynthesis or signaling pathways (Monna 2002; Peng et al. 1999; Spielmeyer et al. 2002). BRs have emerged as a promising focus for the second Green Revolution, owing to their multifaceted roles in plant architecture, grain/fruit quality and biotic/abiotic stress tolerance (Yang et al. 2024a). Many BR mutants have been identified across various species, including *Arabidopsis*, rice, maize, wheat, barley, tomato and pea (Alonso et al. 2024), with most of these mutants being isolated through large-scale mutagenesis. A few naturally occurring alleles have also been identified (Gruszka et al. 2016; Tian et al. 2019), some of which have shown great promise for improving planting density, grain quality and yield (Sakamoto et al. 2006; Tian et al. 2019). BRs were also found to play critical roles in controlling many economically important traits in horticultural crops (Li et al. 2025). Dwarfing is potentially an important trait for woody fruit crops such as grapevine. Grape production is a complex and challenging process that requires careful management of cultural and environmental factors, often by hand. These factors could be minimized or potentially even eliminated by selective breeding to make grape cultivars more amenable to mechanization. Reducing overall plant size is a key step in developing such grape cultivars and could be potentially realized by leveraging dwarfism.

A dwarf mutant grapevine was previously reported. The dwarf mutant vine was derived from a somatic variation in the L1 meristem layer of the grape cultivar *Vitis vinifera* Pinot Meunier (Boss & Thomas 2002). The dwarfism was caused by a point mutation, leading to an amino acid substitution of histidine for leucine at the amino acid position 38, in the DELLA domain in one of the *VviGAI1* (*GIBBERELLIN INSENSITIVE 1*) alleles. *VviGAI1* is homologous to the *GAI* genes *Rht-D1/Rht-B1* in wheat. Mutations in these genes caused dwarfism which was the genetic basis for the wheat green revolution (Boss & Thomas 2002; Franks et al. 2002; Peng et al. 1999). Besides shortened internodes, many tendrils are converted into inflorescences in the *Vvigai1* mutant vines. However, the previously characterized *VvGAI1* mutant in grapevine exhibits an extreme dwarfing phenotype, even in the heterozygous status (Boss & Thomas 2002), and thus has limited commercial applications as a grape cultivar. Mutants involving different domains of *GAI*-like genes were found in many other species. We over-expressed in Arabidopsis a dozen grape versions of known *GAI* mutant alleles reported in wheat, barley, corn, *Brassica*, grape and *Arabidopsis*. The internode length and plant statue were significantly reduced for most of the mutant alleles studied, and as expected, the dwarfing severity varied with specific variants involved (Zhong & Yang 2012). Attempts to explore allelic *GAI* variation for improving plant architecture have been reported in barley (Chandler & Harding 2013).

In this study, we report the discovery and characterization of a new dwarf mutant grapevine. This dwarf vine was derived from breeding crosses made in the Cornell Grapevine Breeding and Genetics Program and showed very different dwarf attributes from the *VviGAI1*-based dwarf mutant. Based on a genetic segregation analysis, we determine that the dwarfism in the mutant vine was controlled by a major locus. Through marker–trait association and bulked RNA-seq data analyses, we concluded that the dwarfing was likely a result of a 9-base pair in-frame deletion in a BR biosynthesis gene, *VviBR6OX1*. We further confirmed the role of this gene by successfully creating a similar dwarf vine phenotype through CRISPR/Cas9-based gene knockout. Additionally, we discovered one other *VviBR6OX* gene, *VviBR6OX2,* whose disruption significantly intensified the dwarfism in conjunction with disrupted *VviBR6OX1*. The discovery and elucidation of the genetic control of a BR-based dwarf mutant in this study provide an important genetic resource and knowledge foundation for developing new grape cultivars with modified vine architecture and other BR-related traits to meet various breeding purposes, including for higher-density planting and reducing labor needs for vineyard management in grape production.

## Results

### Progeny segregation analysis of dwarf mutant vines derived from Cornell grape breeding crosses

**Fig. 1:**
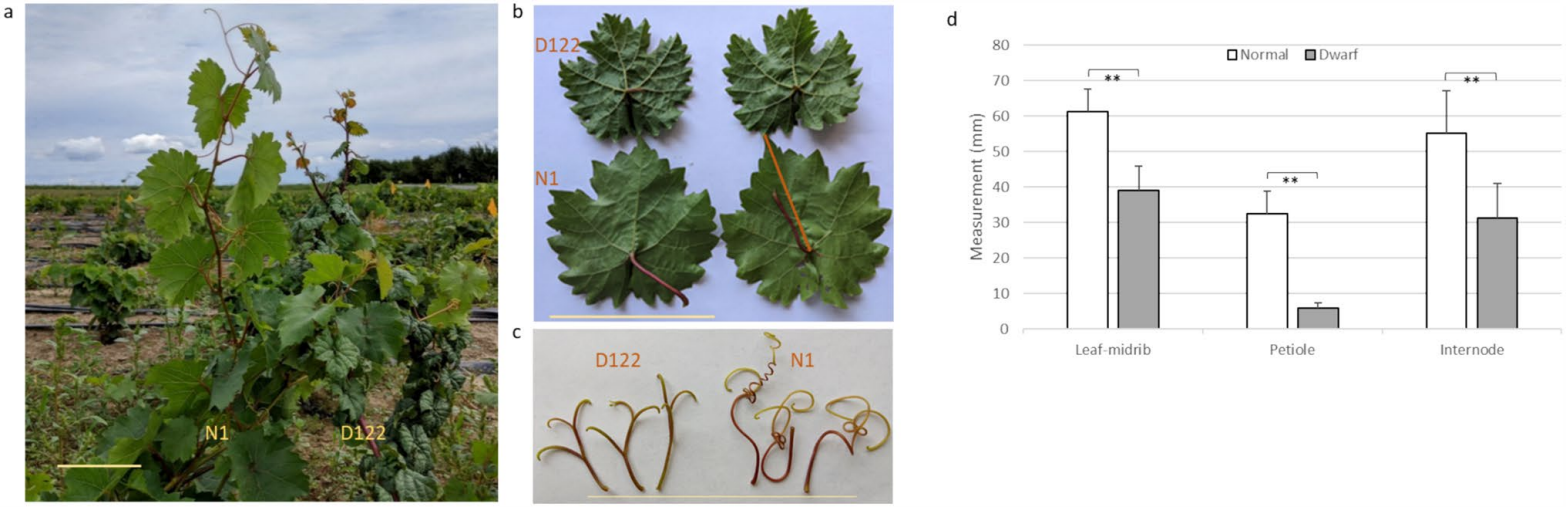
Representative phenotypes of grapevines derived from selfing PI 200569. (**a**) A normal vine (N1) and a mutant dwarf vine (D122) in field. (**b**) Representative leaves. (**c**) tendrils from N1 and D122. The bars in a, b and c are in a scale of 10 cm. (**d**) Trait measurements of leaf midrib length, petiole length, internode length of normal and dwarf vines. Field-grown mutant and normal vines, 20 each, were sampled and measured. One or two branches were cut from each individual vine, and measurements were taken from node 6 to node 12 for leaf midrib length, petiole length and internode length. Leaf midrib length was measured from the leaf tip to the leaf blade base, indicated by the orange line in (**b**). Measurements from individual branches were averaged, and the averaged measurements from individual branches were used for the calculation of means and standard deviations. A total of 38 and 36 branches were measured for normal and mutant vines, respectively. “**” means *p*-value < 0.01 between the two groups in a t-test

Dwarf mutant grapevines with small, dark green leaves, short petioles and short internodes were recovered in the progeny of PI 200569 (Yugoslav 5–24) from the Grapevine Breeding and Genetics Program of Cornell University. The initial observations suggested that the mutant phenotype is controlled by a single recessive locus. To validate this hypothesis and provide further genetic material for investigating genetic control of the mutant trait, we collected and germinated seeds from self-pollinated PI 200569 and developed segregation populations for the trait. In the first batch of 70 seedlings from selfed seeds grown in small 10-cm pots in a greenhouse, 14 exhibited dark green leaves and short vine height. A Chi-square (*X*^2^) goodness-of-fit test confirmed that the segregation of dwarf versus normal individuals fit a 3 normal:1 dwarf Mendelian ratio (*X*^2^ = 0.33). The test validated the previous observation and suggested that dwarfism was likely under control of a single locus. In the subsequent field-grown second batch of 700 additional seedlings, 141 were dwarfs. This segregation ratio was consistent with the observation from the first batch of seedlings, concluding that the dwarfism is a recessive trait and controlled by a single locus. When the vines were seven-month-old, 20 normal-looking and 20 dwarf vines were phenotyped for leaf midrib length (hereafter referred to as leaf size), petiole length and internode length. Significant differences (*P*-value < 0.01) were found between normal-looking and dwarf vines for all three traits. Compared to normal-looking siblings, the dwarf vines showed significantly smaller leaf size (~ 64% of the normal siblings), shorter internodes (~ 56%), shorter petiole length (~ 20%) and less developed tendrils (Fig. 1).

### Identification of *VviBR6OX1* as a candidate gene for the dwarf phenotype

**Fig. 2:**
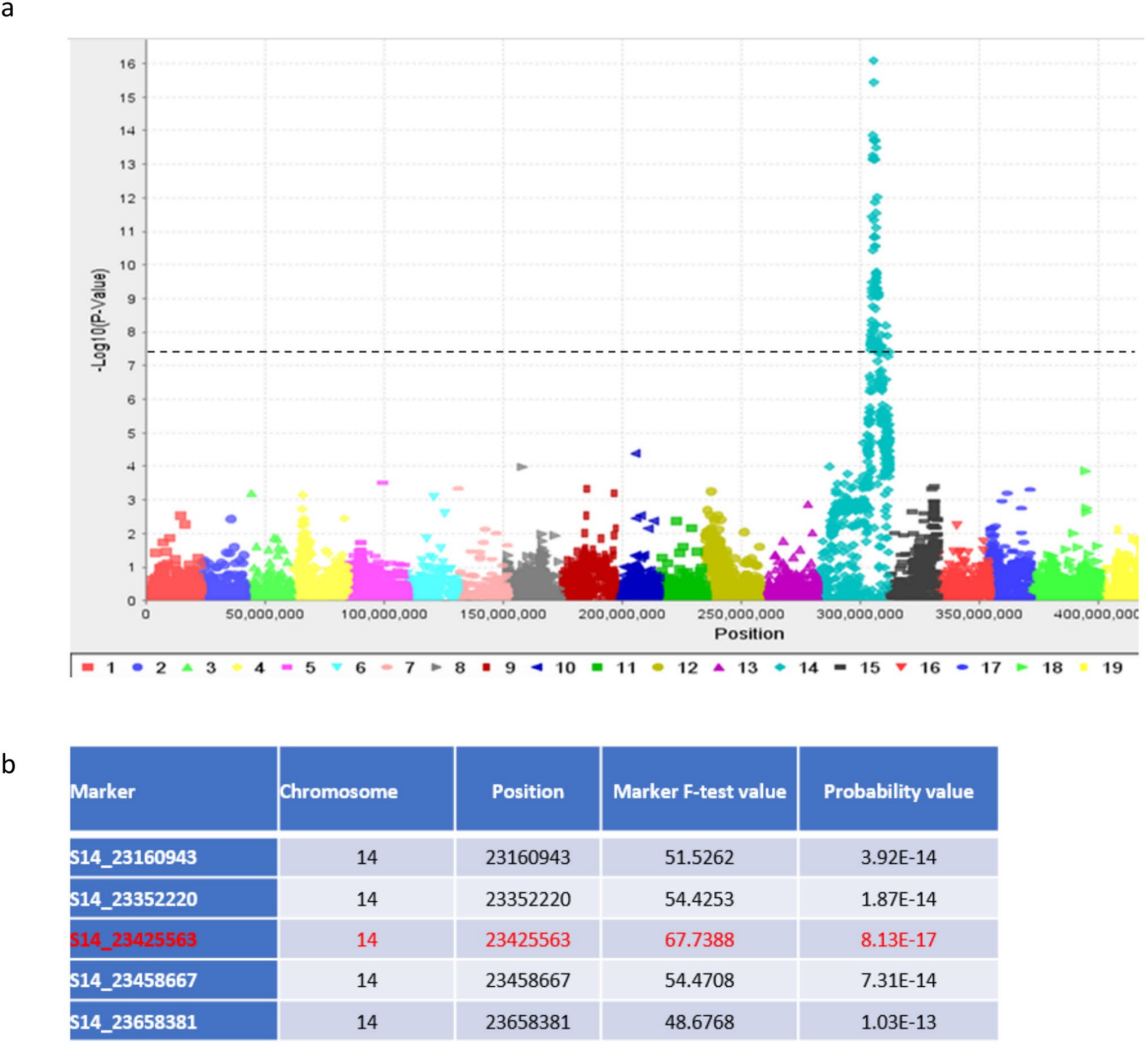
Association of GBS markers with the dwarf mutant phenotype. **a**. A Manhattan plot showing significant marker–mutant trait association on Chromosome 14 (dotted line *P* < 5 × 10^–8^). **b**. Association statistics for the top 5 markers strongly associated with the dwarf mutant phenotype

Genotyping-by-sequencing (GBS) libraries from the first batch of 70 selfed progeny of PI 200569 were constructed and sequenced for mapping the dwarf locus. *V. vinifera* genome PN40024 v2 was used as the reference for SNP calling (Canaguier et al. 2017). A total of 8,355 informative markers were identified, and a Manhattan plot analysis revealed a strong association of the dwarf mutant trait with GBS markers on Chromosome 14 (Fig. 2a). The top 5 markers which were associated with the dwarf mutant trait spanned about 497 kbs (S14_23160943 to S14_23658381) and S14_23425563 appeared to be the strongest one (Fig. 2b).

To refine the candidate interval, we investigated RNA-seq expression profiles of the first batch of 70 selfed progeny grown in the greenhouse. Based on visual evaluation of vine stature, these 70 seedlings were grouped into three groups, with 14 dwarfs labeled as D1–D14, 24 medium ones labeled as M15–M38 and 32 tall ones labeled as T39–T70. We collected three bulk samples of shoot tips and two bulks of leaves from each of the three groups of dwarf, medium and tall vines. Each bulk contained three independent vines, and a total of 15 RNA-seq libraries were constructed (9 from shoot tips and 6 from leaf samples) (Table S1). We aligned the RNA-seq reads to the grapevine reference genome PN40024 v2 and identified many RNA-seq SNPs between the bulk samples of dwarf vines and that of tall vines on Chromosome 14, including those in the region where the putative dwarf locus was located as described above (S14_23160943 to S14_23658381) (Table S2). We identified two RNA-seq SNP markers at the positions of S14_23419894 and S14_23557098 spanning about 137 kbs. This region contained the GBS marker S14_23425563 which was the strongest one associated with the dwarf mutant trait as described earlier. While the two RNA-seq markers were not diagnostic in distinguishing dwarf from medium/tall bulk samples, many RNA-seq SNP markers flanked by S14_23419894 and S14_23557098 were, as illustrated by the fact that the same one allele of the diagnostic markers was present in all dwarf bulk samples but the alternative allele or both alleles were present in medium/tall bulk samples (Table S2).

There were 9 genes annotated in the 137-Kb region (Table 1). Among them, VIT_214s0083g01110 encodes a cytochrome P450 enzyme with high similarity to brassinosteroid-6-oxidase in *Arabidopsis*, rice and other plants (Figure S1). Because of the well-known role of brassinosteroid-6-oxidase in regulating plant development in various species, VIT_214s0083g01110 was the obvious candidate gene for the dwarf mutant phenotype observed. Indeed, a previous study demonstrated that VIT_214s0083g01110, named as *VvBR6OX1*, rescued an extreme dwarfing phenotype in a tomato mutant (dx/dx) when it was transgenically over-expressed (Symons et al. 2006), further suggesting VIT_214s0083g01110 (*VviBR6OX1* hereafter using the grapevine gene nomenclature system (Grimplet et al. 2014)) as the possible genetic cause for the dwarfism observed in this study.

**Table 1:** Candidate genes identified in the 137 Kb QTL region flanked by the RNA-seq markers S14_23419894 and S14_23557098 and their RNA-seq expression levels in dwarf and tall progeny vines of selfed PI 200569

V2 ID^a^	V2 genomic position	V5 ID	V5 genomic position	Annotation	RNA-seq libraries from pooled shoot tissue				RNA-seq libraries from pooled leaf tissue			
					Average expression (RPKM) from dwarf vine shoots (n = 3)	Average expression (RKKM) from tall vine shoots (n = 3)	Fold change (dwarf/tall)	FDR or False Discovery Rate (corrected *p*-value)	Average expression (RPKM) from dwarf vine leaves (n = 2)	Average expression (RPKM) from tall vine leaves (n = 2)	Fold change (dwarf/tall)	FDR or False Discovery Rate (corrected *p*-value)
VIT_214s0083g01090	chr14:23,414,431..23,425,220	Vitvi05_01chr14g20200	chr14:23,332,436..23,341,810	Dimethylguanosine tRNA methyltransferase	5.6824	6.0454	0.9399	1.00	4.1555	5.0087	0.8297	1.00
VIT_214s0083g01100	chr14:23,425,743..23,428,799	Vitvi05_01chr14g20210	chr14:23,341,816..23,345,329	Reversibly glycosylated polypeptide 3	7.9020	3.7101	2.1298	0.00	6.1189	5.8650	1.0433	1.00
VIT_214s0083g01110	chr14:23,435,308..23,438,477	Vitvi05_01chr14g20220	chr14:23,350,926..23,354,799	Brassinosteroid-6-oxidase	8.2111	3.3267	2.4682	0.00	16.7942	5.9575	2.8190	0.02
VIT_214s0083g01120	chr14:23,456,752..23,469,960	Vitvi05_01chr14g20230	chr14:23,372,100..23,374,983	PLC-like protein	0.0000	0.2372	0.0000	0.23	0.0000	0.0431	0.0000	1.00
VIT_214s0083g01130	chr14:23,470,715..23,473,584	Vitvi05_01chr14g20240	chr14:23,386,574..23,390,745	PLC-like protein	0.7553	0.7869	0.9599	1.00	0.8594	1.4713	0.5841	1.00
VIT_214s0083g01140	chr14:23,478,598..23,480,350			B12D protein	0.6038	0.3041	1.9857	0.65	0.7331	0.1878	3.9041	0.69
VIT_214s0083g01150	chr14:23,525,331..23,527,758	Vitvi05_01chr14g20310	chr14:23,431,895..23,434,053	COBRA-like protein family	4.5298	4.2977	1.0540	1.00	2.9593	4.8354	0.6120	1.00
VIT_214s0083g01160	chr14:23,527,885..23,532,718	Vitvi05_01chr14g20320	chr14:23,434,741..23,439,054	COBRA-like protein family	13.2476	8.7905	1.5070	0.00	15.3953	10.1865	1.5113	0.06
VIT_214s0083g01170	chr14:23,552,675..23,569,797	Vitvi05_01chr14g20350	chr14:23,459,091..23,477,233	Mitochondrial substrate carrier family protein	3.8303	4.2782	0.8953	1.00	3.7081	4.5021	0.8236	1.00
VIT_214s0083g01180	chr14:23,573,187..23,576,046	Vitvi05_01chr14g20390	chr14:23,479,904..23,483,772	Protein C9 or f85 homolog	31.0696	33.9903	0.9141	1.00	18.8140	25.2552	0.7450	1.00

^a^V2 and V5 refer to *Vitis vinifera* genome PN40024 v2 and PN40024 v5 (Shi et al. 2023), respectively

To investigate how *VviBR6OX1* might be involved in producing the mutant dwarf phenotype, we conducted an RNA-seq analysis and VIT_214s0083g01110 (brassinosteroid-6-oxidase) was the only DEG in the 137-kb region showing about an increase of over twofold changes in both shoot and leaf bulk tissues between the dwarf and tall samples (Table 1).

**Fig. 3:**
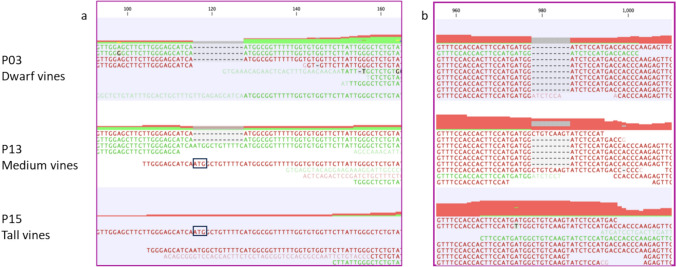
Snapshots of bulked RNA-seq read alignment to the *VviBR6OX1* in the reference genome. **a**. Alignment of the RNA-seq reads involving exon 1 showing a 12-bp deletion in the reads from the bulk samples of dwarf and medium vines. **b**. Alignment of the RNA-seq reads involving exon 4 showing a 9-bp deletion in the reads from the bulk samples of dwarf and medium vines. Reads in green and red were mapped in the forward and reverse directions, respectively, relative to the reference strand. The thickness of red and green blocks reflects the relative numbers of reads for forward and reverse reads, respectively. The thicker the block, the higher the coverage. Gray indicates gaps in reads. The numbers at the top of the panel marked the positions of specific nucleotides in the VviBR6OX1 reference sequence. The starting position “ATG” in exon 1 was marked by a box in reverse reads one each from medium and tall vine samples. The lower read coverage in exon 1 compared to exon 4 is likely due to the 3’ bias introduced by poly(A) selection during RNA-seq library preparation (color figure online)

**Fig. 4:**
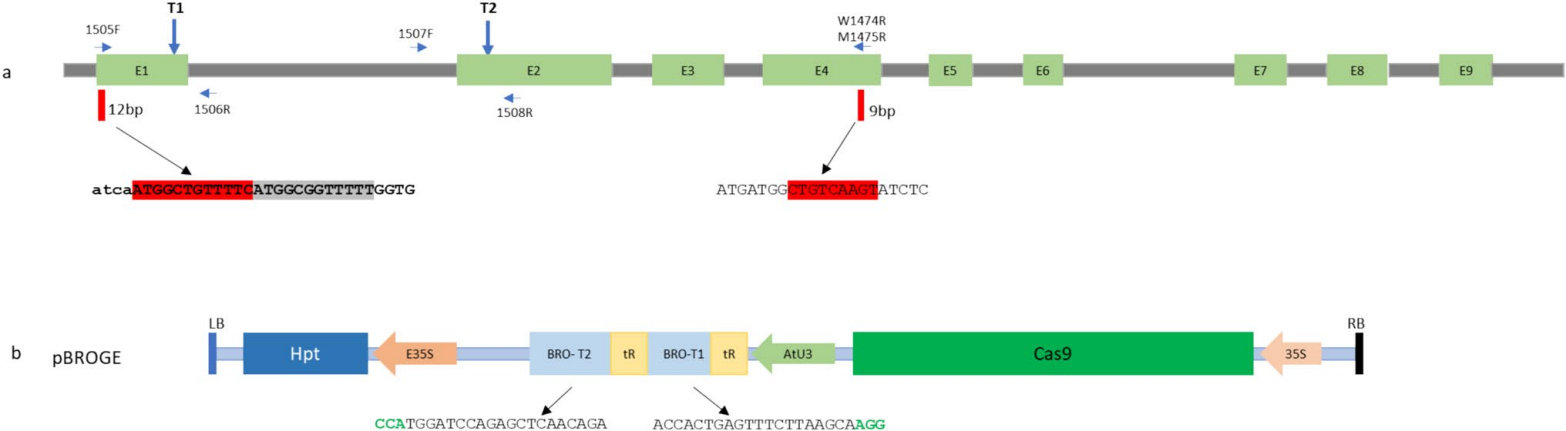
Schematic illustrations of *VviBR6OX1,* Cas9 target sequences and the pBROGE construct. (**a**) *VviBR6OX1* contains 9 exons (green blocks) spanning a ~ 3 kb genomic region. The 12-bp and 9-bp deletions detected in the mutant dwarf vine are marked by red bars in exon 1 and exon 4, respectively. The exact sequences for the two indels are provided and highlighted in red. The 12 bps at the beginning of ORF (highlighted in red) and the next 12 bps (highlighted in gray) in exon 1 are an imperfect tandem repeat. (**b**) The pBROGE construct contains the components of 35S:Cas9, AtU3:tRNA–sgRNA1–tRNA–sgRNA2 and e35S:Hpt in its T-DNA. The two Cas9 target sequences are, respectively, labeled as BRO-T1 and BRO-T2. The PAM sequences are in bold green

We further examined the RNA-seq data of *VviBR6OX1* by aligning its reads against the reference genome and found two indels in the RNA-seq reads of *VviBR6OX1*. The first indel was a 12-bp deletion at the start of the open reading frame or exon 1. This 12-bp sequence showed high similarity to the next 12-bp segment and these two sequence fragments could form an imperfect tandem repeat with 10 out of 12 base pairs being identical (“ATGGCTGTTTTC” vs. “ATGGCGGTTTTT”) (Fig. 3a and 4a). The second indel was a 9-bp deletion in exon 4, resulting in replacing of 4 amino acids “AVKY” with a “D” (Figs. 3b and 4a). All mapped reads in the dwarf vines contained both the 12-bp and 9-bp deletions (Fig. 3), but the reads from the medium vines contained both wild-type and deletion variants. On the other hand, in the tall vines, mainly wild-type reads were detected.

We cloned and sequenced the *VviBR6OX1* gene in PI 500269. Two alleles were identified, one allele with the 12-bp deletion in exon 1 and the 9-bp deletion in exon 4, consistent with the indels identified from the RNA-seq data. The other allele had no such indels (Table S3).

We designed two pairs of primers for genotyping the selfing progenies of PI 500269. One pair of the primers consisted of 1507F and W1474R for wild-type allele and the other pair had 1507F and M1475R, which is specific to the 9-bp deletion (Table S4, Fig. 4a). Genomic PCRs were performed using both sets of primers (Table S4 and Figure S2) for 16 DNA samples of the greenhouse-grown PI 200569 selfing progenies. While band amplifications were observed for 4 medium vines and 8 tall vines with the primer set 1507F/W1474R, no bands were amplified for all four dwarf vines: D3, D8, D13 and D14 (Figure S2). By contrast, the primer set 1507F/M1475R produced clear DNA bands in all four dwarf samples and some of the medium/tall samples. These results suggested that the absence of a wild-type *VviBR6OX1* is the cause of the dwarf phenotype in these dwarf vines.

We further used the primer set 1507F/M1475R to determine the presence or absence of the mutant *VviBR6OX1* allele in the 20 field-grown normal-looking vines which had been phenotyped earlier (Fig. 1d). Among the 20 normal-looking vines, 9 had the wild-type *VviBR6OX1* alleles only (wt/wt) and 11 were heterozygous with both wild-type and mutated alleles (wt/df). We compared the three measured traits between these two groups and observed a significant difference (*p* < 0.05) for petiole length (35 mm for homozygous wild-type vines vs. 31 mm for heterozygous vines), and very small and not significant differences for leaf length and internode length (Figure S3).

### Similar dwarf vine phenotypes recreated through CRISPR–Cas9 editing of *VviBR6OX1*

To further validate the above hypothesis that a mutated *VviBR6OX1* led to dwarfism, we designed a CRISPR–Cas9-based gene-editing construct to knock out the *VviBR6OX1* gene. Two Cas9 target sequences T1 and T2 for targeting the *VviBR6OX1* exon 1 and exon 2, respectively, were incorporated into a synthetic polycistronic tRNA–gRNA gene under the Arabidopsis *U3* promoter (Lowder et al. 2015; Xie et al. 2015) (Fig. 4a and 4b). We transformed the construct into embryogenic callus of *V. vinifera* cv. Scarlet Royal, a widely grown table grape cultivar in California, USA. Once stable transgenic calli were established on hygromycin-containing plates after about 6 months of selection post-initial agro-transformation, representative callus sectors were pooled from seven independent callus plates for genomic DNA extraction and amplicon library construction for both T1 and T2 targets. The amplicon libraries were sequenced through high-throughput amplicon sequencing, and the results showed that the editing efficiencies were very low for the T1 target site, with the highest editing efficiency being only 0.3%. However, T2 exhibited much higher editing rates, which ranged from 0.1% to 99% (Table S5). Poor T1 editing was also confirmed in seven transgenic vines. Five plants had close to 100% editing efficiency at the T2 target site but very low editing efficiency at the T1 site (0.3% or lower) (Table S5).

**Fig. 5:**
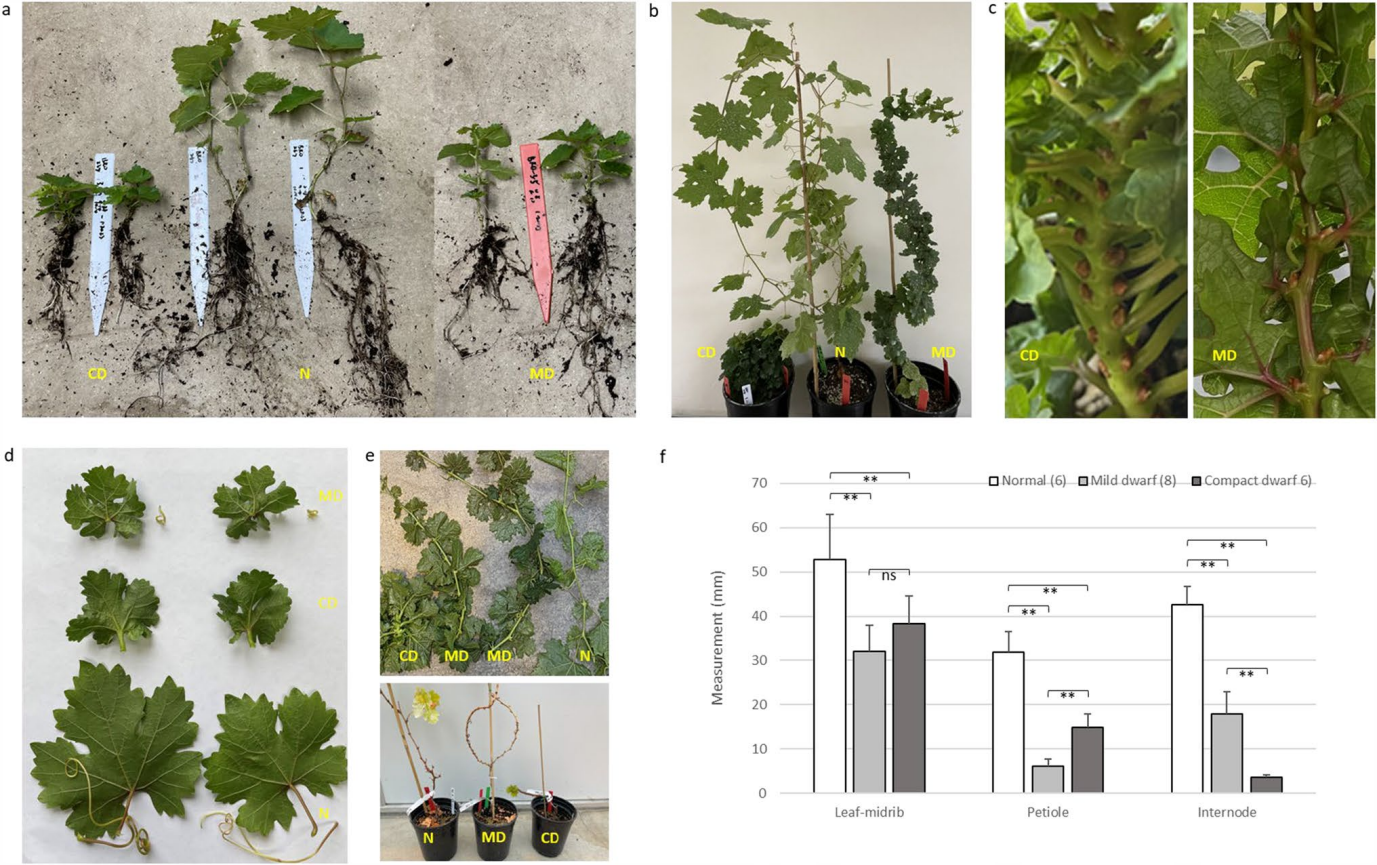
Phenotypes of *VviBR6OX*-edited vines. (**a**) From left to right, three different types of vine stature, two each, of transgenic vines at a young seedling stage of about one month after transplanting in soil from tissue culture medium: compact dwarf (CD), normal (N) and mild dwarf (MD) vines. (**b**) Transgenic vines after 4 to 5 months of growth in a greenhouse. (**c**) Close-up views of a compact dwarf vine (left) and a mild dwarf vine (right) for showing the extremely short internode length but relatively long leaf petioles in the compact vine, and relatively longer internode length and very short leaf petioles in the mild dwarf vine. (**d**) Representative leaves and tendrils from the three types of vines shown in panel b. (**e**) Young shoots of compact dwarf (CD), mild dwarf (MD) and normal-looking (N) vines (top) and defoliated normal (N), mild dwarf (MD) and compact dwarf vines (CD) (bottom). (**f**) Measurements of leaf midrib length, petiole length and internode length of normal-looking vines (n = 6), mild dwarf vines (n = 8) and compact dwarf vines (n = 6). “**” indicates that the *p*-value is less than 0.01 between the two groups in a t-test while “ns” (no statistically significant difference) means that the *p*-value is more than 0.05

We transplanted over 60 vines from tissue culture boxes into 10-cm pots, and these transgenic vines were later transplanted into one-gallon pots and grown in a greenhouse. More than half of these transgenic vines exhibited dwarf phenotypes with shortened internodes, reduced leaf petiole length and smaller dark green leaves. The root systems of the dwarf vines appeared relatively normal when we repotted these vines from 10-cm pots into one-gallon pots (Fig. 5a). Interestingly, two distinct types of dwarf vines were observed. While both had significant reduction in internode length, leaf petiole length and leaf size, one type was more severe than the other (Fig. 5a–f). We referred to the vines with less reduction of internode length as mild dwarf vines and the ones with more severe reduction as compact dwarf vines. The average internode length, petiole length and leaf size for mild dwarf vines were 42.1%, 19.1% and 60.5% of the control (normal-looking transgenic vines), respectively. In contrast, the average internode length, petiole length and leaf size for compact dwarf vines were 8.5%, 46.3% and 72.4% of the control. The compact dwarf vines had much shorter internode length than mild dwarf vines (Fig. 5c, e and f). However, petiole length was much less reduced in the compact dwarf vines than the mild dwarf vines (Fig. 5c, d and f).

The gene knockout experiment, along with the association mapping data, provided convincing evidence that *VviBR6OX1* was responsible for the natural mutant dwarf phenotype reported in this study. However, it was puzzling why two types of dwarf vines were created in the knockout experiment.

### Compact dwarf vines likely resulted from simultaneous editing of two *BR6OX* genes

Because two different dwarf phenotypes were so frequently observed across our independently edited vines, these phenotypes cannot plausibly be attributed to rare off-target editing events or to position effects associated with random transgenic integration sites. We suspect that a similar target site from a different gene was affected. We blasted the *VviBR6OX1* sequence against the reference genome and realized the presence of an additional *BR6OX* gene in grape genomes. This additional *BR6OX* gene, named *VviBR6OX2,* was previously reported in an expression study of BR-related genes (Parada et al. 2022). The protein sequences of VviBR6OX1 and VviBR6OX2 (VIT_201s0011g00190) share about 72.8% identity (Figure S1). The pBROGE construct we built for this study had two gRNA target sequences for editing *VviBR6OX1.* While T1 had only about 75% match with its corresponding sequence in *VviBR6OX2* (5 mismatches out of 20 bps), T2 was 100% identical between *VviBR6OX1* and *VviBR6OX2* (Figure S4). We suspect that *VviBR6OX2* might have been edited at the T2 site. We examined the *VviBR6OX2* editing with the same set of callus samples and the vines used for initial evaluation of the *VviBR6OX1* editing, with the *VviBR6OX2*-specific primer set (1749/1750) (Table S5). The two pooled callus samples with high editing efficiency for *VviBR6OX1* also showed comparably high editing efficiency for *VviBR6OX2* at the same T2 target site. Similarly, the 5 transgenic vines with high editing efficiency for *VviBR6OX1* also showed high editing efficiency for *VviBR6OX2*.

To determine the edited changes of *VviBR6OX1* and *VviBR6OX2* and their associations with dwarf mutant phenotypes, we sampled small leaf sectors from 46 transgenic vines, constructed and sequenced amplicon libraries spanning the T2 target site in both *VviBR6OX1* and *VviBR6OX2* genes, and analyzed the T2 editing efficiencies (Table 2). We did not evaluate editing profiles at the T1 target site due to its extremely low editing efficiency in both transgenic callus and transgenic vine samples, as reported earlier (Table S5). For many transgenic dwarf vines, multiple samples from different parts of a vine at the same or different vine developmental stages were analyzed (Table 2). Among the 46 transgenic vines studied, 7 showed compact dwarf phenotypes, 21 were mild dwarf, and 15 appeared to be normal-looking (Table 2). Three out of the 46 vines developed shoots with more than one phenotype (Table 2 and Figure S5). Specifically, both BR18 and BR51 had mild (M) and compact dwarf (C) shoots while BR53 had normal (N) and mild dwarf (M) shoots. Many edited variants were detected in transgenic vines for both *VviBR6OX1* and *VviBR6OX2* genes but only those with 1% or more of total mapped reads were reported as major editing variants in this study (Tables 2, S6 and S7). In all mild and compact dwarf vines, the *VviBR6OX1* gene was almost completely edited, with the average editing percentages ranged from 99.65% to 100% (Table 2). Most of these dwarf vines had only 3 or fewer major edited variants and altogether they accounted for more than 90% of the total mapped reads. Mild dwarf vines BR16, BR24 and BR53M had 4 to 7 major edited variants, but the two top edited variants together still accounted for more than 85% of the total mapped reads in these vines.

**Table 2:** Transgenic vines and their phenotypes and editing profiles described in this study

Transgenic vine ID	Phenotype	Leaf samples for amplicon sequencing		*VviBR6OX1* editing					
		No. of samples	No. of sampling time points	InDelSub^a^ (%)	No. variants^b^ (%)	Most edited variant type^c^	InDelSub (%) for most edited variant^d^	Second most edited variant type	InDelSub (%) for second most edited variant
BR1	Mild dwarf	2	2	99.95	2	10 bp del	50.43–51.29	6 bp del	47.20–48.07
BR4	Mild dwarf	1	1	99.70	1	"A" in	96.67		
BR5	Mild dwarf	1	1	100.00	2	19 bp del	51.90	"G" del	46.39
BR6	Mild dwarf	2	2	99.65	1	"A" in	97.73–98.15		
BR8	Mild dwarf	5	2	99.85	3	28 bp del	42.21–45.96	"A" in	39.62–45.94
BR9	Mild dwarf	1	1	100.00	2	4 bp del	49.35	3 bp del	49.29
BR10	Mild dwarf	2	2	99.70	1	"G" del	98.19–98.28		
BR11	Mild dwarf	2	1	99.90	1	6 bp del	98.85–98.89		
BR12	Mild dwarf	1	1	100.00	2	10 bp del	49.58	6 bp del	49.05
BR13	Mild dwarf	4	2	99.90	1	"G" del	98.67–98.81		
BR16	Mild dwarf	8	3	99.90	4	37 bp del	42.35–53.67	6 bp del	24.85–32.91
BR17	Mild dwarf	2	2	99.85	2	"AT" del	49.72–50.87	"A" in	47.34–47.85
BR18M^e^	Mild dwarf	1	1	99.80	2	41 bp del	55.82	"A" in	42.64
BR19	Mild dwarf	2	2	100.00	1	6 bp del	98.29–98.50		
BR20	Mild dwarf	8	3	100.00	2	8 bp del2	48.89–51.76	"AT" del	47.75–49.34
BR23	Mild dwarf	1	1	100.00	2	8 bp del1	63.42	"AT" del	34.33
BR24	Mild dwarf	4	2	99.90	4–7	"A" del	50.41–70.38	6 bp del	0.00–24.80
BR25	Mild dwarf	2	2	99.90	1	"G" del	98.42–98.78		
BR45	Mild dwarf	1	1	100.00	2	"AT" del	50.75	"T" del	47.16
BR46	Mild dwarf	1	1	100.00	1	"G" del	98.24		
BR50	Mild dwarf	2	2	100.00	1	78 bp del	98.31–99.34		
BR51M^e^	Mild dwarf	2	1	100.00	1	37 bp del, 2 m	99.82–99.08		
BR53M^e^	Mild dwarf	2	2	100.00	3–7	"A" in	54.366–68.488	16 bp del	0.00–20.48
BR54	Mild dwarf	1	1	100.00	2	8 bp del2	60.12	"AT" del	38.07
BR2	Compact dwarf	1	1	99.70	1	"A" in	98.36		
BR3	Compact dwarf	4	2	100.00	2	41 bp del	56.89–59.09	"GA" del, 1 m	39.83–41.55
BR7	Compact dwarf	3	2	100.00	3	11 bp del2	47.85–50.07	"A" del	30.86–34.40
BR14	Compact dwarf	14	3	99.90	2	7 bp del1	49.92–54.80	"A" in	42.29–47.79
BR18C^e^	Compact dwarf	1	1	100.00	2	41 bp del	56.52	9 bp del2	42.18
BR30	Compact dwarf	5	2	100.00	1	"G" del	98.76–98.81		
BR31	Compact dwarf	5	2	100.00	3	41 bp del	48.39–54.49	24 bp del	33.92–35.68
BR32	Compact dwarf	5	3	99.80	2–3	8 bp del2	47.42–50.80	"A" in	44.73–50.93
BR51C^e^	Compact dwarf	4	2	99.95	1	37 bp del, 2 m	98.74–98.88		
BRN33	Normal	2	2	1.75	0				
BRN42	Normal	1	1	12.10	1	7 bp del1	11.94		
BRN47	Normal	1	1	13.20	2	7 bp del1	7.19	"A" in	5.80
BR53N^e^	Normal	1	1	99.80	3	"A" in	45.02	"A" to "T" ms ( a.a. substitution of D for V)	30.39
BRO2671	Normal	1	1	0.00	0				
BRO2672	Normal	1	1	0.10	0				
BRO2674	Normal	1	1	1.00	**0**				
BRO2677	Normal	1	1	0.60	0				
BRO51	Normal	1	1	4.10	**0**				
BRO621	Normal	1	1	1.50	0				
BRO661	Normal	1	1	0.70	0				
BRO671	Normal	1	1	0.00	0				
BRO741	Normal	1	1	0.10	0				
BRO742	Normal	1	1	0.20	0				
BRO743	Normal	1	1	0.10	0				
BRO744	Normal	1	1	0.10	0				

Transgenic vine ID	*VviBR6OX2* editing					
	InDelSub (%)	No. variants (%)	Most edited variant type	InDelSub (%) for most edited variant	Second most edited variant type	InDelSub (%) for second most edited variant
BR1	73.15	4–13	"A" in	12.83–19.74	8 bp del2	< 0.01–17.38
BR4	96.80	6	8 bp del1	54.04	"A" in	11.14
BR5	99.80	6	8 bp del4	34.79	"T" in1	19.99
BR6	91.80	10–16	8 bp del 1	52.07–59.19	6 bp del1	7.05–10.74
BR8	97.78	4–7	5 bp del1	38.11–51.70	21 bp del3	< 0.01–48.29
BR9	50.9	5	7 bp del1	18.39	31 bp del3	7.99
BR10	95.2	9 -11	4 bp del3	12.37–22.71	8 bp del4	15.26–16.87
BR11	97.8	6	"T" del	49.56–55.84	"A" in	16.06–19.53
BR12	90.9	5	51 bp del	48.43	5 bp del2	11.85
BR13	72.9	3 -9	6 bp del1	16.26–24.69	"T" del	0.66–30.04
BR16	94.2	6–8	20 bp del	34.39–44.37	"TC" del, 1 m	9.61–22.58
BR17	92.15	6–8	"AT" del	41.52–55.05	11 bp del2	9.24–25.15
BR18M^e^	99.4	6	"T" in1	43.65	56 bp del	30.20
BR19	82.4	4–6	"T" del	35.15–51.58	"A" in	5.92–16.24
BR20	88.26	3–10	8 bp del4	51.74–59.20	"A" in	3.16–38.53
BR23	99.6	5	"A" del, 2 m	50.52	"A" in	26.65
BR24	98.1	9–12	"A" del	2.61–20.77	6 bp del1	1.78–21.93
BR25	98.5	8–15	"A" in	13.03–31.95	47 bp del2	0.00–22.88
BR45	99.6	2	2 bp del	56.29	"A" in	41.88
BR46	100.00	7	4 bp del1	51.49	4 bp del2	32.77
BR50	99.9	3–12	11 bp del2	1.15–53.58	6 bp del1	< 0.01–31.82
BR51M^e^	98.3	2–8	"T" del	49.51–50.24	9 bp del	7.53–10.99
BR53M^e^	99.9	3	45 bp del2	55.60–59.46	"A" in	29.46–39.04
BR54	88.3	6	8 bp del4	50.72	2 bp del	13.47
BR2	99.8	2	8 bp del4	54.48	"A" in	44.34
BR3	99.5	2	"T" in1	82.11–90.76	indel comp2	6.95–12.38
BR7	99.83	4	"A" in	33.73–40.90	6 bp del1	37.11–38.51
BR14	100.00	2	21 bp del3	49.03–53.06	8 bp del6	31.32–47.97
BR18C^e^	99.5	2	"T" in1	51.01	7 bp del1	46.82
BR30	99.8	2	9 bp del1	51.59–63.40	"A" in	34.65–46.73
BR31	99.7	3–5	"T" in1	42.38–53.66	6 bp del1	35.26–39.11
BR32	99.8	2	"AT" del	48.80–50.43	"A" in	46.95–48.00
BR51C^e^	99.93	3	"T" del	47.42–50.01	"A" in	0.02–48.57
BRN33	1.20	0				
BRN42	0.00	0				
BRN47	Not determined	Not determined				
BR53N^e^	99.80	3	45 bp del2	76.22	"A" in	13.12
BRO2671	0.10	0				
BRO2672	0.10	0				
BRO2674	1.20	0				
BRO2677	0.60	0				
BRO51	3.30	0				
BRO621	0.70	0				
BRO661	0.40	0				
BRO671	0.20	0				
BRO741	0.20	0				
BRO742	0.30	0				
BRO743	0.10	0				
BRO744	0.10	0				

^a^InDelSub% (short for insertion–deletion–substitution) was generated by Cas-Analyzer. For plants with multiple samples, the average was presented. The editing variant analysis was conducted in a 100 bp window with the PAM site located at the 34 bp position for *VviBR6OX1* and 47 bp for *VviBR6OX2*

^b^Number of major edited variants with an editing efficiency of 1% or more. When multiple variants were present in the same sample or across different samples, the range of the number of variants was indicated

^c^**In, del and m** means insertion, deletion and mismatch, respectively. For examples, **"A" in** means an insertion of a nucleotide "A"; and **37 bp de, 2 m** means a deletion of 37 base pairs and presence of additional 2 mismatches. If more than one type of deletion with the same base pairs was present among the samples, a number was added to distinguish different deletion types such as **del1** and **del2**. See Table S6 for examples

^d^The percentage of an editing variant was calculated by the number of all reads with the variant signature indel divided by the number of total mapped reads to the reference

^e^Two different phenotypes were found for the edited vines BR18, BR51 and BR53. A suffix "C," "M" or "N" was added to the IDs of these vines for indicating a "compact dwarf," "mild dwarf" and "normal" phenotype, respectively

The editing profiles for *VviBR6OX2* gene varied significantly among different types of dwarf vines. The 7 compact dwarf vines and the compact dwarf branches from BR18 and BR51 (i.e., BR18C and BR51C) all had an editing percentage of 95.5% or above (Table 2). Six of them had only two variants, and three others had 3–5 edited variants with the top two variants accounting for more than 70% of the total mapped reads. In comparison, 20 mild dwarf vines and the mild dwarf branches from BR18 and BR51 (i.e., BR18M and BR51M) had 5 or more major edited variants and 7 of them had 10 or more. The *VviBR6OX2* editing percentage accounted for by the top two edited variants in most of these vines were about 20–30% of total mapped reads, although few exceptions were present. This was in a sharp contrast to what were observed for the compact dwarf vines in which most had only 2 major edited variants of *VviBR6OX2* with more than 70% edited changes accounted for.

We compared the editing profiles for both compact dwarf and mild dwarf shoots that were simultaneously present in both BR18 and BR51 (Table 2 and Table S7). In both BR18 and BR51, all amplicon sequencing samples showed almost 100% editing for both *VviBR6OX1* and *VviBR6OX2*, regardless of whether the DNA samples were from compact or mild dwarf shoots*.* Both compact and mild dwarf shoots had one or two major edited variants for *VviBR6OX1*. However, the mild dwarf shoots in both BR18 and BR51 contained more edited *VviBR6OX2* variants than the compact dwarf shoots. Specifically, BR18C had only two edited *VviBR6OX2* variants while BR18M had 6. BR51C had 2 or 3 edited *VviBR6OX2* variants and BR51M had 7 to 8 (Table S7). Furthermore, we identified two groups of vines with identical editing profiles for *VviBR6OX1*, but with different editing profiles for *VviBR6OX2* and distinctive phenotypes (Tables 2 and S7). One group includes three vines: BR2, BR4 and BR6. They all have only one major *VviBR6OX1* editing variant with an “A” insertion. The compact dwarf vine, BR2, has only two *VviBR6OX2* variants but the two mild dwarf vines, BR4 and BR6, have multiple *VviBR6OX2* editing variants (6 for BR4, 10–16 for BR6). The other group includes five vines, BR10, BR13, BR25, BR30 and BR46, with identical *VviBR6OX1* editing of a “G” deletion. The compact dwarf vine BR30 in the group has only two *VviBR6OX2* variants. In contrast, the four mild dwarf vines in the group have 3 to 15 *VviBR6OX2* variants. These observations consistently support that *VviBR6OX2* editing variants, when present as dominant alleles, would increase the severity of the dwarf phenotypes and were responsible for the compact dwarf phenotypes observed in this study.

Among the 16 normal-looking vines/shoots, nine had less than 1% editing for both *VviBR6OX1* and *VviBR6OX2* (Table 2). Six vines had editing percentages ranging between 1% and 13.2% for *VviBR6OX1* and 0 to 3.3% for *VviBR6OX2*. However, BR53N, referring to a normal-looking shoot from BR53, had both *VviBR6OX1* and *VviBR6OX2* almost 100% edited (Table 2, Table S7 and Figure S5). A closer review of the editing profile in BR53N revealed an interesting editing variant of *VviBR6OX1* which had a substitution of “A” for “T” resulting in a missense mutation (G*A*T to G*T*T) and replacement of an aspartic acid with a valine at the amino acid position 83. This missense mutation accounted for 30.39% of the total mapped *VviBR6OX1* reads (Table 2 and Table S7). Apparently, this missense mutation, although accounted for only 30.39% of the total *VviBR6OX1* reads, could largely perform the full *VviBR6OX1* function and support normal vine development. It was also interesting to note that, although *VviBR6OX2* was 100% edited in BR53N, the vine could still develop quite normally, suggesting that *VviBR6OX2* was not a primary determinant of dwarfism.

### Expression patterns and indel variants of *VviBR6OX* genes in PI 500269 selfing progeny and *Vitis germplasm*

A previous study revealed that both *VviBR6OX* genes had almost undetectable expression in most tissues, except in immature seeds and green berries (*VviBR6OX1*) or immature and mature seeds (*VviBR6OX2*) (Parada et al. 2022). We investigated the RNA-seq expression profiles of *VviBR6OX1* and *VviBR6OX2* in the shoots and leaves of the dwarf, medium and tall vines/seedlings of PI 500269 selfing progeny (Table S1). *VviBR6OX1* was expressed in both shoots and leaves, and its expression level appeared higher in leaves than in shoots, especially in dwarf vines (Figure S6). Interestingly, dwarf vines had higher *VviBR6OX1* expression than the medium and tall vines in both shoot and leaf tissues, as reported earlier (Table 1). The 9-bp in-frame deletion likely abolished the *VviBR6OX1* function and reduced BR levels, which in turn leads to upregulation of the genes in the BR synthesis pathway including *VviBR6OX1*. This is in agreement with the well-established feedback regulation of BR biosynthetic genes (He et al. 2005; Tanaka et al. 2005). We analyzed some of our in-house RNA-seq data from unrelated projects and found *VviBR6OX1* also expressed in leaves, shoots, roots and developing berries from different *Vitis* species and genotypes. Consistently higher levels of *VviBR6OX1* expression than that of *VviBR6OX2* were reported for almost all grape tissues, including berries, leaves, shoots, roots and tendrils, from the microarray-based genome-wide transcriptomic atlas (Table S8) (https://bar.utoronto.ca/efp_grape/) (Fasoli et al. 2012; M. Wang et al. 2014). In contrast, for the 28 embryogenic callus RNA-seq libraries, *VviBR6OX2* expression was surprisingly high and comparable to that of *VviBR6OX1* in shoots and leaves, while *VviBR6OX1* expression was very low in the same callus samples.

To detect whether the *VviBR6OX1* indels observed in PI 200569 are present in other grapevines, we blasted the *VviBR6OX1* gene sequence from the reference genome against the grapevine genome databases available on NCBI (https://blast.ncbi.nlm.nih.gov/). We found that some sequenced grapevine genomes, such as “Tannat,” shared the same 12-bp deletion at the N-terminal (Da Silva et al. 2013) as observed in the PI 200569 accession. We further examined the presence of the 12-bp and 9-bp indels in 21 V*. vinifera* genomes listed on the grape genomics website (https://www.grapegenomics.com/). Over half of these sequenced *V. vinifera* genomes contained at least one *VviBR6OX1* allele with the identical 12-bp deletion, including those from “Riesling,” “Zinfandel,” “Chardonnay” and “Pinot noir” (Table S9). Additionally, we analyzed hundreds of grape genome sequences available on NCBI (https://www.ncbi.nlm.nih.gov/datasets/genome/) and revealed that 82 out of 390 surveyed accessions contained this same deletion. However, we did not observe in any of the genomes analyzed an occurrence of the second in-frame 9-bp deletion as reported in this study.

The presence of the 12-bp in-frame deletion, which removes four amino acids at the N-terminal, appeared not critical for *VviBR6OX1* function. For instance, the cultivar “Malbec” has both alleles of *VviBR6OX1* with the 12-bp deletion (Table S9), but it is not dwarf. We also surveyed the two *VviBR6OX1* indels in several wild *Vitis* species whose genomes are available on the grape genomics website (https://www.grapegenomics.com/). None of the 10 wild species contained the 12-bp deletion (Table S9). Moreover, we did not observe any occurrences of the 12-bp deletion in four sequenced genomes of *Vitis vinifera* ssp. *Sylvestris,* the dioecious wild ancestor of cultivated *V. vinifera*, and two *Muscadinia rotundifolia* genomes (Table S9). These data suggest that the 12-bp deletion was a unique feature of cultivated *V. vinifera* accessions. In addition to the widespread 12-bp deletion at the beginning of the *VviBR6OX1* ORF, we also identified other indels in the *VviBR6OX1* coding region in a few cultivars (Table S9). For example, one copy of *VviBR6OX1* in “Chardonnay” has a 2-bp insertion in exon 3, while one copy of *VviBR6OX1* in “Merlot” has a 2-bp deletion in exon 1 and a 1-bp deletion in exon 4 (Table S9). It is expected that some dwarf vines would be found among their selfed progenies.

## Materials and Methods

### PI 200569 and its selfed progeny populations

PI200569 (Yugoslav 5–24) was maintained as a parental line in the Cornell grapevine breeding program and as a genetic resource in the USDA Agricultural Research Service Grape Germplasm Repository located in Geneva, New York. Selfed seeds from PI200569 were collected in 2015 and 2019. These seeds were germinated in 2016 and grown as seedlings in greenhouses. The 2016 seedlings were scored as dwarf, medium and tall when they had about 10 leaves in a greenhouse. The 2019 selfed seeds were germinated in February 2020 and transplanted into the McCarthy Nursery in Geneva, NY in the Spring. Vines in the field were phenotyped for internode length, leaf petiole length and leaf midrib length (referred to as leaf size) in September 2020. For phenotyping, 20 plants in each of two vine stature groups (dwarf and normal) were assessed. Briefly, one or two branches were collected from individual vines, and measurements were taken from nodes 6 to 12. Measurements from each branch were averaged, and these averages were used to calculate means, standard deviations and for the t-test. A total of 38 branches were measured for normal vines and 36 branches for the dwarf mutants. The 20 normal vines were also genotyped for the presence or absence of the dwarf *VviBR6OX1* allele with the primer set 1507F/M1475R (Figure S3 and Table S4).

### GBS libraries, RNA-seq libraries and data analyses

Leaf tissues were collected from individual vines and used for genomic DNA extraction using the Qiagen DNeasy Plant Kit. Genotyping-by-sequencing (GBS) libraries were constructed using the methylation-sensitive restriction enzyme ApeKI (NEB) and sample-specific barcoded adapters (Elshire et al. 2011). Libraries were pooled and sequenced at the Cornell Biotechnology Resource Center (BRC). Raw reads were demultiplexed by barcode and quality filtered, then collapsed into unique sequence tags. Tags were aligned to the *Vitis vinifera* reference genome PN40024 v2, and SNP genotypes were called using the reference-based TASSEL-GBS pipeline (Glaubitz et al. 2014). SNPs were filtered to remove loci with high missingness and low minor allele frequency, and individuals with excessive missing data were excluded prior to association testing.

For RNA-seq, the same 70 seedlings were grouped by stature into dwarf, medium and tall classes. Bulked samples were collected from shoot tips (three bulks per class) and leaves (two bulks per class), with three independent vines per bulk (total 15 RNA-seq libraries: 9 shoot tip and 6 leaf libraries; Table S1). Total RNA was extracted using the Plant Spectrum RNA Kit (Sigma). Libraries were prepared following our previous protocol (Yang et al. 2015) and sequenced at Cornell BRC as single-end 1 × 100 bp, unstranded. RNA-seq reads were quality filtered and aligned to PN40024 v2 using STAR (Dobin et al. 2013). Gene-level counts were generated using featureCounts (Liao et al. 2014). Differential expression between dwarf and non-dwarf bulks was tested using DESeq2 (Love et al. 2014). Genes were considered differentially expressed using an FDR-adjusted *p*-value threshold of padj < 0.01 (primary; padj < 0.05 also evaluated), together with a minimum fold change cutoff of ≥ 1.5 × (|log2FC|≥ log2(1.5) ≈ 0.585). RNA-seq–derived SNP markers were identified from the RNA-seq alignments by comparing allele states between dwarf and non-dwarf bulks, focusing on Chromosome 14, including the candidate interval supported by the GBS association signal (S14_23160943–S14_23658381; Table S2). Candidate indels/variants in prioritized genes (e.g., *VviBR6OX1*) were examined by visual inspection of read alignments in Qiagen CLC Genomics Workbench.

Marker–trait association analyses were conducted in TASSEL v5.0 using a mixed linear model (MLM) framework (Bradbury et al. 2007), with marker genotype fitted as a fixed effect. Population structure was modeled using the first five principal components (PCs) as covariates, and relatedness was accounted for using a kinship term in the MLM framework; efficient MLM implementations in TASSEL (including compressed MLM/P3D concepts) are widely used for genome-wide scans (Zhang et al. 2010). Association *p*-values were computed per marker and visualized as Manhattan plots using –log10(p) across genomic positions (Fig. 2a), and the most strongly associated loci were ranked by *p*-value and summarized (Fig. 2b).

### Primer design and genomic PCR

Primer W1474R spanning the 9-bp deletion was for specifically amplifying the wild-type *VviBR6OX1* gene and primer M1475R was specifically for the mutant *VviBR6OX1* allele with the 9-bp deletion (Fig. 4 and Table S4). The expected size of 1507F/W1474R or 1507F/M1475R amplicons was 961 bps. For genomic PCRs, 5 ng of genomic DNA was used in a 20 µl reaction with GoTaq DNA polymerase (Promega). The PCR conditions were as follows: an initial denaturation at 95 °C for 3 min, followed by 35 cycles of 95 °C for 30 s, 58 °C for 30 s and 72 °C for 60 s, with a final extension at 72 °C for 5 min. A 5 µl aliquot of the PCR products was examined on a 1% agarose gel.

### Editing construct

Two Cas9 target sites for *VviBR6OX1* were selected (Fig. 4). The two target sequences, separated by tRNA sequences, were synthesized by GenScript and cloned into an entry vector (*YPQ141B*) under the control of the Arabidopsis *U3* promoter (*141B-BRO*). The binary vector (*pBROGE*) was assembled through gateway cloning using the *pMDC32* destination vector, *YPQ167* with Cas9 and *141B-BRO* (Fig. 4) (Lowder et al. 2015).

### Transformation and phenotypic characterization of transgenic vines

*Agrobacterium* strain *EHA105* harboring the *pBROGE* construct was used to transform embryogenic callus of *V. vinifera* cv Scarlet Royal, following our previous protocol (Yang et al. 2024b). Over 60 transgenic vines were generated in this study. Six representative normal-looking plants (Normal), eight mild dwarf plants (mild dwarf) and six compact dwarf plants (compact dwarf) (~ 8.5 months after embryo induction) were phenotyped in a manner similar to that described for phenotyping field-growing vines. Measurements were averaged for each branch, and the averaged values were used to calculate means and standard deviations. The mean differences were statistically determined using a T-test.

### Amplicon library construction, sequencing and data analysis

Primers were designed to cover the two target sites in *VviBR6OX1* (Fig. 4 and Table S4). To assess the editing efficiency of the *VviBR6OX1* gene in transgenic callus, genomic DNA was extracted from seven pools of hygromycin-resistant transgenic calli. Amplicon libraries were constructed using primers 1505/1506 for T1 and 1507/1508 for T2, following our previous protocol (Yang et al. 2024b). These DNA samples were also assessed for *VviBR6OX2* editing efficiency later with primer set (1749/1750). The amplicon data were analyzed using the Cas-Analyzer with the following parameters (50 bp for compare range, minimum frequency > 1 and 5 bp for WT marker) (http://www.rgenome.net/cas-analyzer/) (Park et al. 2017).

To evaluate the editing efficiencies for *VviBR6OX1* and *VviBR6OX2* in different transgenic vines, amplicon libraries were constructed using either extracted leaf genomic DNA samples by following the same protocol as described for the callus genomic DNA or leaf tissue directly for the first round of PCR. We used the PHIRE Plant Direct PCR kit (www.Thermofisher.com) for the second method. A small sector of leaf tissue was collected from a young leaf of each plant, minced and resuspended in 30 µl of dilution buffer. Half microliter resuspension was used for a 20 µl PCR reaction, which contained 10 µl of PCR mix, 0.5 µl of resuspended leaf tissue, 0.1 µl of 100 µM forward primer, 0.1 µl of 100 µM reverse primer and 9.3 µl of H₂O. The PCR conditions were as follows: 98 ˚C for 5 min, followed by 38 cycles of 98 ˚C for 5 s, 62 ˚C for 5 s, 72 ˚C for 20 s and a final extension at 72 ˚C for 1 min. The PCR products (5 µl) were examined on a 1% agarose gel. The remaining PCR products were purified using 1.2 volumes of Ampure beads (Beckman) and then eluted in 20 µl of water. The second round of PCR for barcoding is the same as described in the first method.

## Discussion

### Phenotypic manifestation and genetic control of grapevine dwarf mutants

Here we report the first BR-related dwarf mutant vine which was found in breeding crosses from the Cornell University grapevine breeding program. Through association mapping, RNA-seq analysis and a germplasm survey, we determined that a 9-bp deletion in *VviBR6OX1*, a brassinosteroid (BR) synthesis gene, was responsible for the observed dwarfism. The BR dwarfing effect was found to vary with different traits in this study, with a severe reduction in leaf petiole length but a relatively mild reduction in internode length and leaf size.

Compared to the previously discovered dwarf mutant vine which resulted from a point mutation in the DELLA domain of the *VviGAI1* gene (Boss & Thomas 2002), the dwarf mutant vine reported in this study showed much less pronounced dwarfing characteristics. The *GAI1* mutant showed an extreme dwarfing phenotype, even in the heterozygous state. In contrast, the BR mutant did not show apparent dwarfing when the mutated *VviBR6OX1* was in a heterozygous status (Figure S3). The dwarfing effect became much more apparent when the mutated *VviBR6OX1* was in a homozygous state (Fig. 1a), but it was still much less severe than a heterozygous *VviGAI1* mutant (Figure S7a). Genetic background was not a major factor in contributing to this sharp difference because similar severe dwarfing characteristics were found when the mutated *VviGAI1* was introgressed into different cultivars (Arro et al. 2024). In addition to the overall dwarfing characteristics such as shortened internode length and dark green leaf, *VviGAI1* mutant vines also exhibited a conversion of tendrils into inflorescences (Boss & Thomas 2002). This characteristic was, however, not seen in transgenic vines with the same *Vvigai1* mutant copy (Arro et al. 2019), nor in similar GA mutants in *Arabidopsis* or in *VviBR6OX1* mutants. Furthermore, seeds carrying a *VviGAI1* DELLA point mutation usually required a special GA_3_ treatment for germination (Boss & Thomas 2002). In some cases, GA₃ treatment is ineffective and embryo rescue is required for obtaining seedlings (Chatbanyong & Torregrosa 2015). Homozygous BR mutant dwarf vines exhibited less developed tendrils and reduced fertility, but they still produced viable seeds. Despite the observation in this study that two batches of selfed PI 500269 seeds resulted in approximately 20% homozygous dwarf vines, less than what would be expected of 25%, the impact of mutated *VviBR6OX1* on seed germination is not fully investigated. While both gibberellins (GA) and brassinosteroids (BR) play positive roles in controlling seed germination and a possible cross talk between BR and GA in co-regulating seed germination in rice was reported (Xiong et al. 2021), BR, compared to GA, likely plays a secondary role in seed germination in grapevine. The less affected seed germination in *VviBR6OX1* mutant seeds could also be a result of the presence of an active *VviBR6OX2*, which was known to express at a relatively high level in immature and mature seeds (Parada et al. 2022) and might play a role in seed germination.

### Roles of *VviBR6OX1* and *VviBR6OX2* in causing dwarfism

*VviBR6OX* belongs to the CYP85A family of cytochrome P450 monooxygenases. Like many other cytochrome P450s, the CYP85A family often has multiple homologs in the same species (Kim et al. 2005; Nomura et al. 2005). For example, the Arabidopsis genome contains two CYP85A genes. Mutations in *Cyp85A1* resulted in a mild dwarf phenotype, while mutations in *Cyp85A2* caused more pronounced dwarfism, darker green leaves and reduced fertility. However, double mutants (*cyp85a1cyp85a2*) exhibited severe brassinosteroid (BR) deficiency and were extremely dwarf (Castle et al. 2005; Kim et al. 2005; Nomura et al. 2005). In addition to *VviBR6OX1,* an additional *BR6OX* gene, *VviBR6OX2*, was previously reported in an expression study of BR-related genes (Parada et al. 2022). When we designed two gRNAs for editing *VviBR6OX1* to confirm its role in grapevine dwarfism, *VviBR6OX2* was inadvertently not considered. It turned out that the gRNA for targeting the T1 site in the construct did not work for either of the *VviBR6OX* genes, but the gRNA for editing the T2 site was very effective for both. This unintentional editing of *VviBR6OX2* provided us an opportunity to investigate the roles of both *BR6OX* genes in grapevine dwarfism.

Two distinct types of dwarf grapevines were found in the transgenically edited vines. One was mild and the other was very compact (Fig. 5 and Table 2). *VviBR6OX1* was edited at the T2 target site in all mild dwarf and compact dwarf vines. All compact dwarf vines, however, had both *VviBR6OX* genes almost 100% edited at the T2 site. This suggests that knocking out *VviBR6OX2* was a key cause for leading a vine to show a compact dwarf phenotype when the *VviBR6OX1* was also completely knocked out in the vine. One intriguing finding to be explained was that more than half mild dwarf vines were also almost 100% edited for both *VviBR6OX* genes (Table 2). Further review of the editing profiles of these vines revealed that mild and compact dwarf vines appeared to differ in the presence of the numbers of major edited *VviBR6OX2* variants. The mild dwarf vines with almost 100% *VviBR6OX2* edited contained multiple edited variants of *VviBR6OX2*, each with relatively lower percentages of total mapped reads, while most compact dwarf vines showed only two major edited variants with much higher percentages of total mapped reads (Table 2 and Table S7). We hypothesized that early-stage knockout of *VviBR6OX2* would intensify dwarfism of a *VviBR6OX1* knockout vine. When *VviBR6OX2* was edited and disrupted at an early vine developmental stage, almost all cells would carry few dominant, similar edited alleles. These cells would have very limited or no BRs available due to the disruption of both *VviBR6OX* genes, resulting in a compact dwarf phenotype. On the other hand, if *VviBR6OX2* was not bi-allelically edited in most cells at an early developmental stage in a vine, the cells with unedited *VviBR6OX2* might still be able to provide some BR functions. Once a vine passes a critical developmental stage such as the onset of internode elongation, the dwarfing enhancing effect of knocking out *VviBR6OX2* may not be observed, even if additional *VviBR6OX2* alleles are edited, resulting in close to 100% editing in mature leaf samples. Clearly this hypothesis needs to be further examined.

BR53N was a unique case offering some important insights into how *VviBR6OX1* and *VviBR6OX2* contributed to the observed dwarfism. BR53N shoot appeared normal, but it had both *VviBR6OX1* and *VviBR6OX2* completely edited (Table 2 and Table S7). Upon a scrutiny of the editing profiles, we found that the second most edited *VviBR6OX1* variant was a missense editing, which led to the substitution of the amino acid of aspartic acid for valine. Most likely, this missense editing allele, even though accounted for only 30.39% of all the edited *VviBR6OX1* reads, could still largely support the normal function of *VviBR6OX1* for the vine development. Very interestingly, in the original young vine BRO53 from which BR53N was derived, about 17.65% of *VviBR6OX1* was found with the missense mutation. However, the original BRO53 vine was observed as mild dwarf (Table S7). Apparently, there was a dosage threshold for the missense edited allele to fully compensate the function of a normal *VviBR6OX1* allele. Another important finding was that *VviBR6OX2* was completely edited in the vine, but the vine did not show any apparent dwarfing characteristics (Figure S5), suggesting that *VviBR6OX2* was not a determinant factor for causing vine dwarfing, although it could intensify the dwarfing effect of *VviBR6OX1*. A similar finding of two redundant BR genes with one playing a leading role was previously reported in *Arabidopsis* in which two *CYP85A* (*BR6OX*) genes (*CYP85A1* and *CYP85A2*) exhibited functional redundancy. Mutations in *CYP85A2* resulted in a mild dwarf phenotype, while mutations in *CYP85A1* showed no growth defects; only the double mutant exhibited severe dwarfism (Nomura et al. 2005). In contrast to *Arabidopsis*, in species such as cucumber*,* rice and maize, which have a single *BR6OX* gene or one highly expressed gene, a single mutant of the respective gene could result in severe growth defects and sterility (Gruszka et al. 2016; Hong et al. 2002; Makarevitch et al. 2012; Wang et al. 2017).

### Potential of *VviBR6OX1 *and* VviBR6OX2 in improving* grapevine architecture

Brassinosteroids (BRs), although discovered relatively recently, have become a focal point in the second Green Revolution due to their critical roles in stress resistance and regulation of agronomic traits such as plant architecture, grain yield, fruit quality and abiotic stress response (Yang et al. 2024a). In crops like rice, wheat and maize, mutations that disrupt BR synthesis or signaling have been leveraged for development of short-statured cultivars suitable for higher-density planting with enhanced grain yields and no additional nitrogen input (Morinaka et al. 2006; Sakamoto et al. 2006; Song et al. 2023). These successes strongly supports modification and optimization of BR signaling/synthesis as a promising strategy for future field crop cultivar development (Yang et al. 2024a). In many apple dwarf rootstocks, BR levels were reduced due to the inhibited expression of the BR rate-limiting synthetase gene *MdDWF4* by the transcription factor MdWRKY9 (Zheng et al. 2018). However, such a cultivar improvement strategy has not been well explored for the improvement of plant architecture in grapevine.

Both *VviBR6OX1* and *VviBR6OX2* contributed to dwarfism observed in this study, but *VviBR6OX1* played the key role. This was not a surprise because *VviBR6OX2* was primarily detected in callus and had very low levels in other examined tissues. Therefore, *VviBR6OX2*, compared with *VviBR6OX1,* is not likely a suitable target for modifying vine architecture. Nevertheless, further studies are needed to better understand the functional divergence between *VviBR6OX1* and *VviBR6OX2* and their tissue expression specificities. In *Arabidopsis*, two *CPY85A* genes have been demonstrated with different expression patterns and catalytical capabilities in BR synthesis. *AtCYP85A1* is only able to catalyze the synthesis of castasterone (CS) while *AtCYP85A2* is able to synthesize both CS and BL (brassinolide) which is more biologically active (Castle et al. 2005; Nomura et al. 2005). Given that BL was not detected in all examined grape tissues and *VviBR6OX1* was the main expressed *BR6OX* gene in these tissues (Table S8) (Parada et al. 2022), we speculate that *VviBR6OX1*, like *AtCYP85A1*, may not be able to catalyze the synthesis of BL. Whether *VviBR6OX2* is capable of synthesizing BL, similar to the function of *CYP85A3* in tomato or *CYP85A2* in *Arabidopsis*, is unknown. Since *VviBR6OX2* was expressed at a much high level in embryogenic callus as found in this study, it would be interesting to determine whether BL could be detected in the callus tissue. Understanding the functional role of *VviBR6OX2* in specific grapevine cell types could provide valuable insights into its contribution to vine early development and how it interacts with *VviBR6OX1*.

There are many examples of exploring mutational changes in BR genes for creating desirable stature of plants with varying dwarfing. For example, in rice, *d2* mutants with a specific amino acid substitution exhibited milder phenotypes compared to those with a premature stop codon or a substitution at a more conserved residue (Hong et al. 2002). Another example is that in *Brachypodium distachyon*, the *Bdbrd1-1* heterozygotes were about 30% shorter than wild-type plants, with homozygous mutants being even smaller (Xu et al. 2015). Furthermore, in barley, several missense mutations in the *BR6OX* gene *HvDWARF* have been identified through TILLING and chemical mutagenesis (Gruszka et al. 2011, 2016). These mutations lead to a range of dwarfing phenotypes, from mild to severe, for further use in breeding. In this study, heterozygous BR mutant vines (wt/df), compared to homozygous wild-type vines (wt/wt), appeared to have shorter leaf petiole length (Figure S3). It is conceivable that we could modify the expression level or create different types of *VviBR6OX1* mutants to produce vines with varying degrees of dwarfism. The presence of two different phenotypes in the same vines in this study suggested that the effects of BRs were localized, confirming the previous conclusion that BRs cannot be transported over long distance (Symons et al. 2007). Such localization property of BRs could provide an opportunity to modify BR-related abiotic stress resistance traits in rootstocks without concerning potential BRs’ impact on scion traits in grapevines.

This study primarily focused on the influences of mutated *VviBR6OX* genes on several vine architecture traits, but BRs were shown to impact many other economically important traits in horticultural plants, including grapevines (Li et al. 2025). For example, exogenous epibrassinolide application increased endogenous BR levels, enhanced anthocyanin accumulation and berry coloration, and promoted softening via upregulation of anthocyanin biosynthetic genes and alterations in pectin and cell wall-related processes in grapes (Liu et al. 2025). Another example is that exogenous application of epibrassinolide can alleviate oxidative damage, improve photosynthetic capacity and regulate carbon and nitrogen assimilation, thus improving the tolerance of grapevine to drought stress (Zeng et al. 2024). These BR-related trait improvement opportunities could potentially be realized through enhancing expression of *VviBR6OX* and other BR-related genes via gene editing.

In summary, we discovered, characterized and validated *VviBR6OX1* as the primary genetic factor responsible for a BR-related dwarf mutant which was first reported in grapevine. We further demonstrated that *VviBR6OX1* could be modified via CRISPR–Cas9 with a high editing efficiency to produce dwarf vines. This work provides an important step in leveraging BR genes, as has been demonstrated in many field crops, to develop new grapevine cultivars with improved plant architecture and other horticultural traits to meet various breeding purposes, such as for higher-density planting in grape production. The knowledge reported here may also benefit other woody fruit species, such as apples, in which dwarfing rootstocks and scions are highly desirable.

## Competing interest

The authors declare no competing interests.

## Supplementary Information

Below is the link to the electronic supplementary material.

Supplementary file1 (PPTX 47128 kb)

Supplementary file2 (XLSX 61 kb)

## Data Availability

The 15 RNA-seq libraries were deposited at NCBI under the BioProject ID PRJNA1430808.
