# Supplementary material for: First brassinosteroid-based dwarf mutant discovered and characterized in grapevine
Source: Theor Appl Genet. 2026 May 4;139(5):147. doi: 10.1007/s00122-026-05225-6 (PMC13139300; doi:10.1007/s00122-026-05225-6)
Supplement: Supplementary file 1 — Supplementary file1 (PPTX 47128 kb) [file 122_2026_5225_MOESM1_ESM.pptx]

## Slide 1
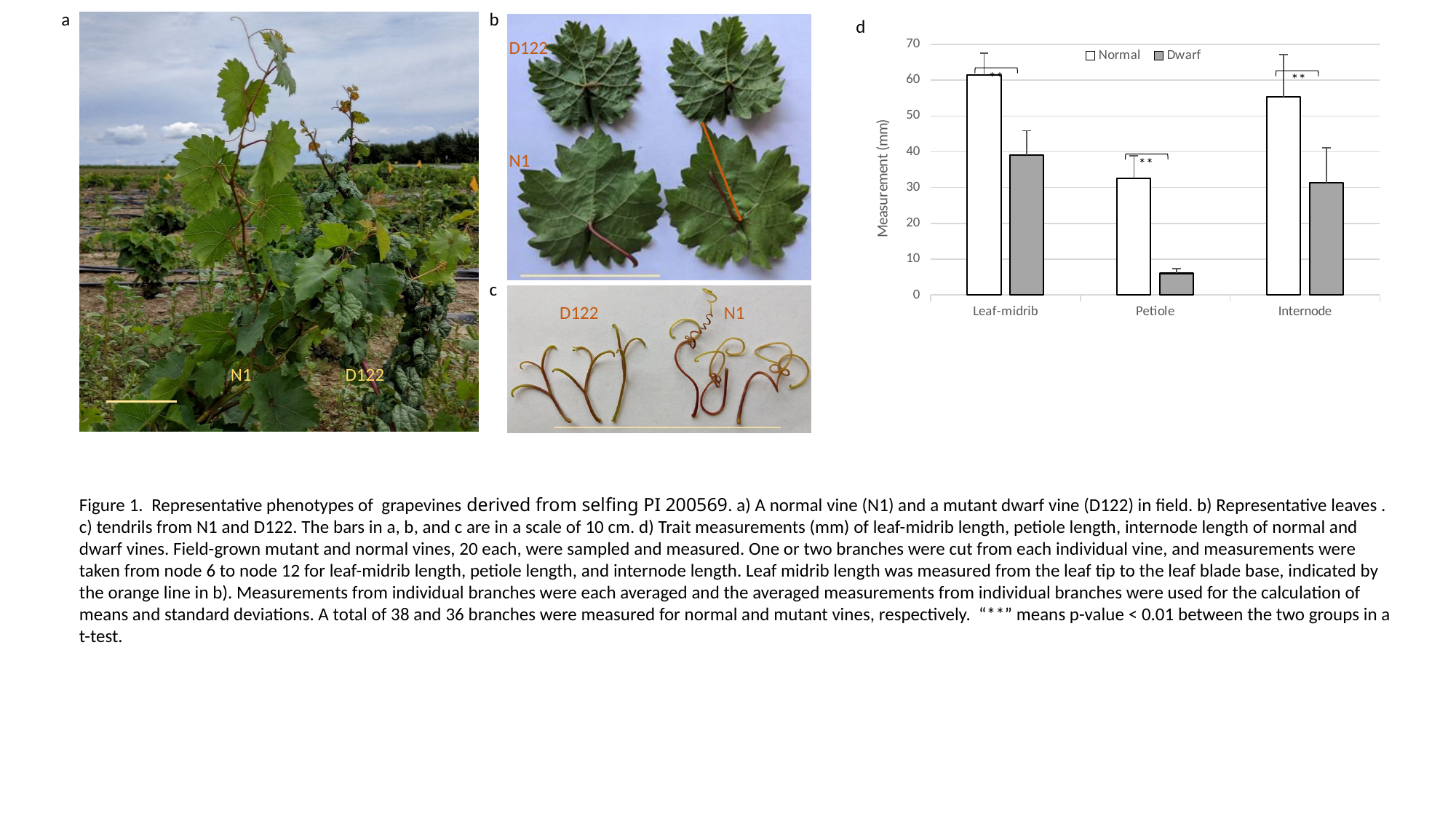

a
b
d
N1
D122
D122
N1
### Chart
| Category | Normal | Dwarf |
|---|---|---|
| Leaf-midrib | 61.39 | 39.05 |
| Petiole | 32.51 | 6.04 |
| Internode | 55.31 | 31.4 |
**
**
**
c
D122
N1
Figure 1. Representative phenotypes of grapevines derived from selfing PI 200569. a) A normal vine (N1) and a mutant dwarf vine (D122) in field. b) Representative leaves . c) tendrils from N1 and D122. The bars in a, b, and c are in a scale of 10 cm. d) Trait measurements (mm) of leaf-midrib length, petiole length, internode length of normal and dwarf vines. Field-grown mutant and normal vines, 20 each, were sampled and measured. One or two branches were cut from each individual vine, and measurements were taken from node 6 to node 12 for leaf-midrib length, petiole length, and internode length. Leaf midrib length was measured from the leaf tip to the leaf blade base, indicated by the orange line in b). Measurements from individual branches were each averaged and the averaged measurements from individual branches were used for the calculation of means and standard deviations. A total of 38 and 36 branches were measured for normal and mutant vines, respectively. “**” means p-value < 0.01 between the two groups in a t-test.

## Slide 2
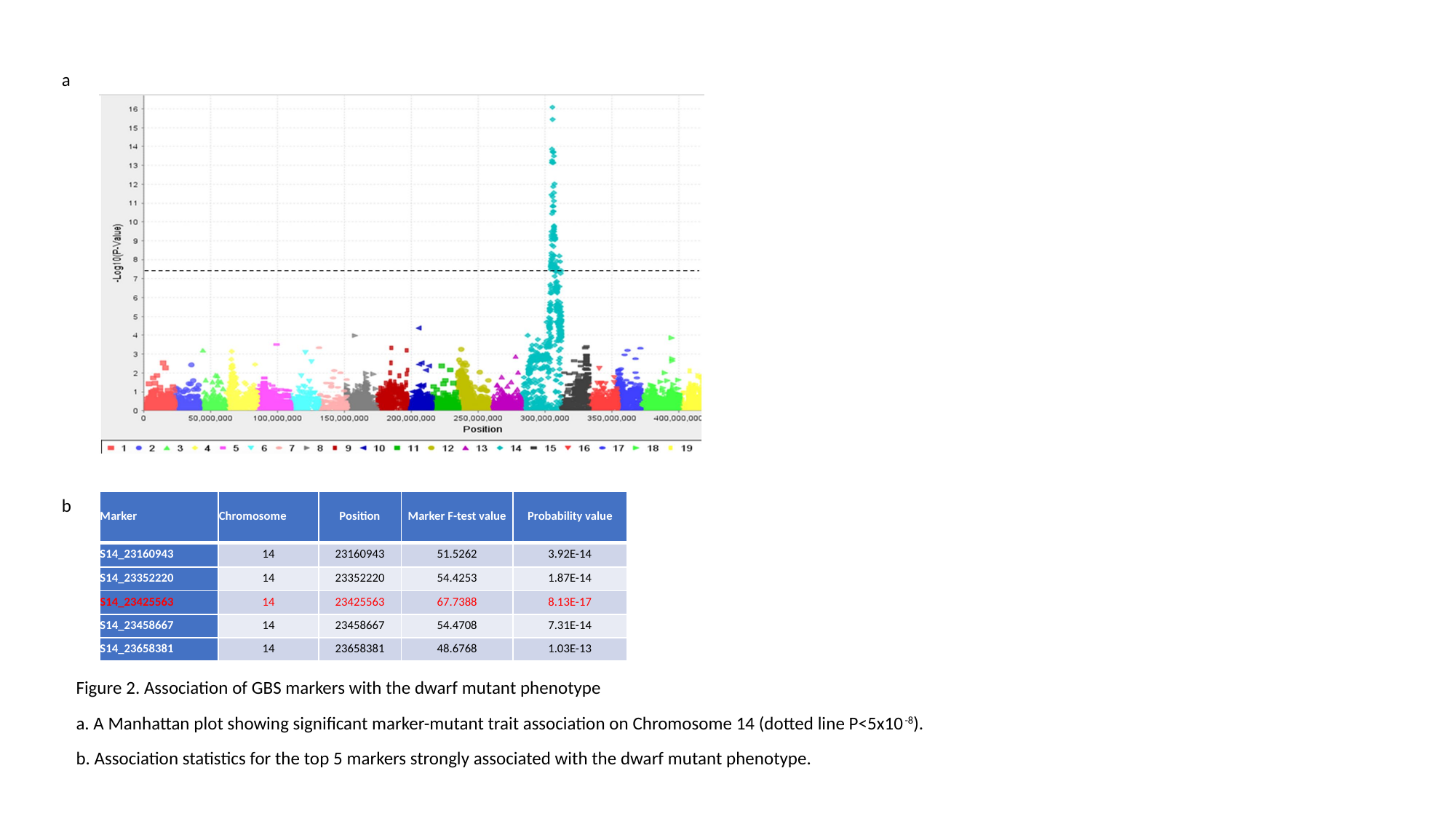

a
b
| Marker | Chromosome | Position | Marker F-test value | Probability value |
| --- | --- | --- | --- | --- |
| S14\_23160943 | 14 | 23160943 | 51.5262 | 3.92E-14 |
| S14\_23352220 | 14 | 23352220 | 54.4253 | 1.87E-14 |
| S14\_23425563 | 14 | 23425563 | 67.7388 | 8.13E-17 |
| S14\_23458667 | 14 | 23458667 | 54.4708 | 7.31E-14 |
| S14\_23658381 | 14 | 23658381 | 48.6768 | 1.03E-13 |
Figure 2. Association of GBS markers with the dwarf mutant phenotype
a. A Manhattan plot showing significant marker-mutant trait association on Chromosome 14 (dotted line P<5x10^-8^).
b. Association statistics for the top 5 markers strongly associated with the dwarf mutant phenotype.

## Slide 3
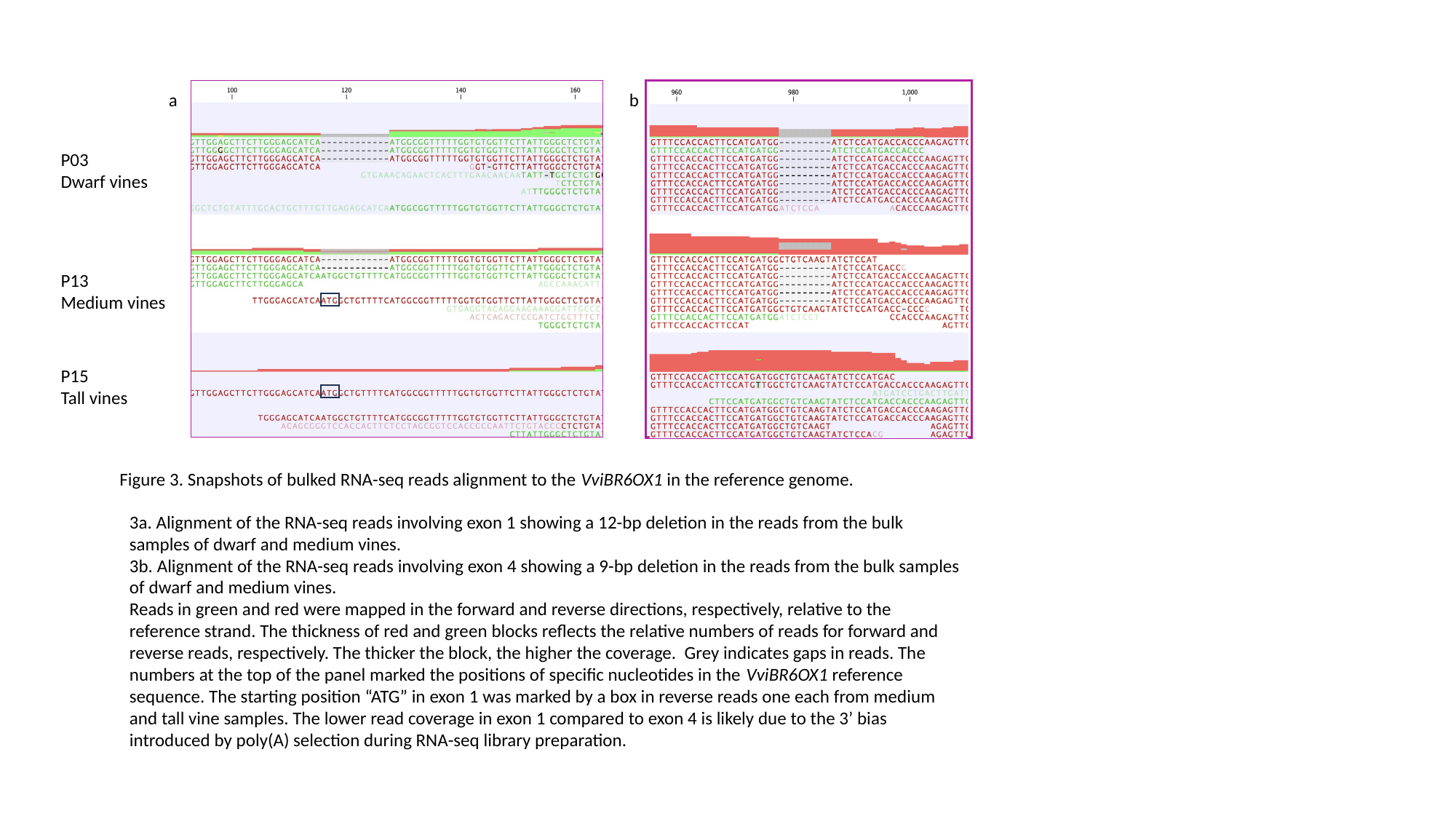

a
b
P03
Dwarf vines
P13
Medium vines
P15
Tall vines
Figure 3. Snapshots of bulked RNA-seq reads alignment to the VviBR6OX1 in the reference genome.
3a. Alignment of the RNA-seq reads involving exon 1 showing a 12-bp deletion in the reads from the bulk samples of dwarf and medium vines.
3b. Alignment of the RNA-seq reads involving exon 4 showing a 9-bp deletion in the reads from the bulk samples of dwarf and medium vines.
Reads in green and red were mapped in the forward and reverse directions, respectively, relative to the reference strand. The thickness of red and green blocks reflects the relative numbers of reads for forward and reverse reads, respectively. The thicker the block, the higher the coverage. Grey indicates gaps in reads. The numbers at the top of the panel marked the positions of specific nucleotides in the VviBR6OX1 reference sequence. The starting position “ATG” in exon 1 was marked by a box in reverse reads one each from medium and tall vine samples. The lower read coverage in exon 1 compared to exon 4 is likely due to the 3’ bias introduced by poly(A) selection during RNA-seq library preparation.

## Slide 4
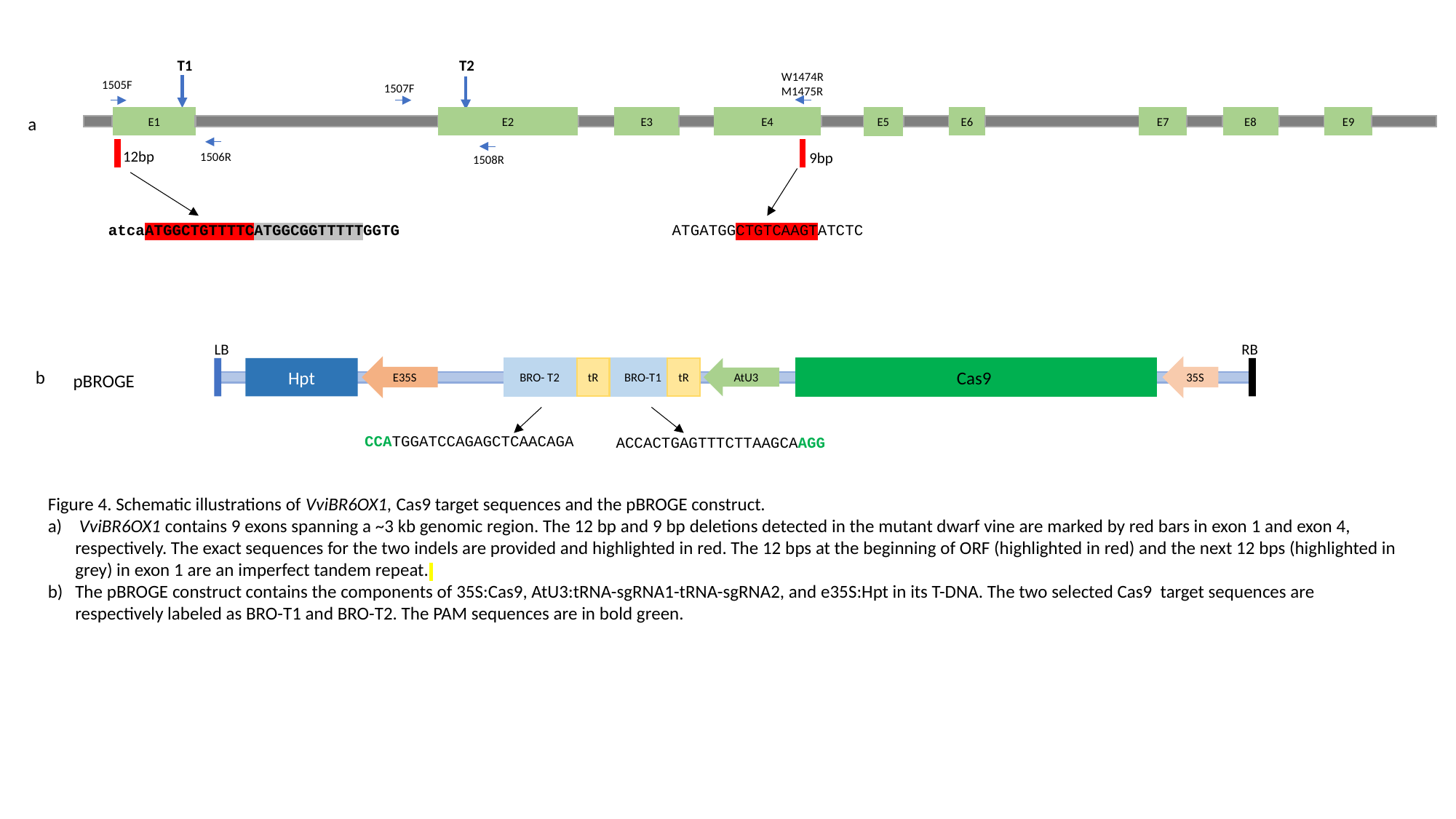

T1
T2
W1474R
M1475R
1505F
1507F
E5
E1
E2
E3
E4
E6
E7
E8
E9
a
12bp
9bp
1506R
1508R
atcaATGGCTGTTTTCATGGCGGTTTTTGGTG
ATGATGGCTGTCAAGTATCTC
RB
LB
Hpt
E35S
BRO- T2
tR
BRO-T1
tR
AtU3
Cas9
35S
b
pBROGE
CCATGGATCCAGAGCTCAACAGA
ACCACTGAGTTTCTTAAGCAAGG
Figure 4. Schematic illustrations of VviBR6OX1, Cas9 target sequences and the pBROGE construct.
 VviBR6OX1 contains 9 exons spanning a ~3 kb genomic region. The 12 bp and 9 bp deletions detected in the mutant dwarf vine are marked by red bars in exon 1 and exon 4, respectively. The exact sequences for the two indels are provided and highlighted in red. The 12 bps at the beginning of ORF (highlighted in red) and the next 12 bps (highlighted in grey) in exon 1 are an imperfect tandem repeat.
The pBROGE construct contains the components of 35S:Cas9, AtU3:tRNA-sgRNA1-tRNA-sgRNA2, and e35S:Hpt in its T-DNA. The two selected Cas9 target sequences are respectively labeled as BRO-T1 and BRO-T2. The PAM sequences are in bold green.

## Slide 5
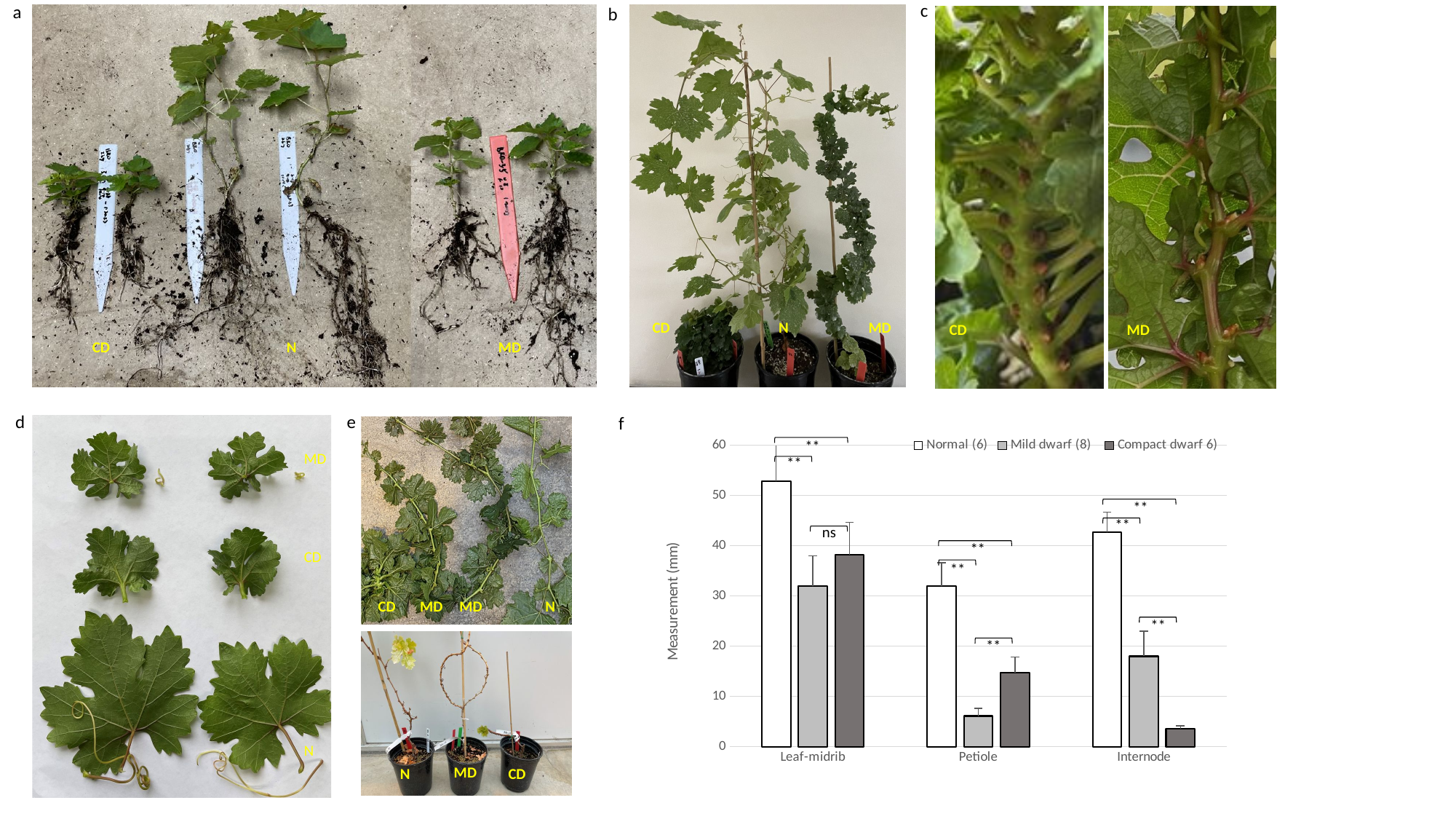

c
CD
MD
a
CD
N
MD
b
CD
N
MD
d
e
MD
CD
N
CD
MD
MD
N
MD
N
CD
f
### Chart
| Category | Normal (6) | Mild dwarf (8) | Compact dwarf 6) |
|---|---|---|---|
| Leaf-midrib | 52.85714285714286 | 31.997023809523814 | 38.25 |
| Petiole | 31.952380952380953 | 6.098214285714285 | 14.785714285714286 |
| Internode | 42.666666666666664 | 17.982142857142858 | 3.619047619047619 |
**
**
**
**
ns
**
**
**
**

## Slide 6
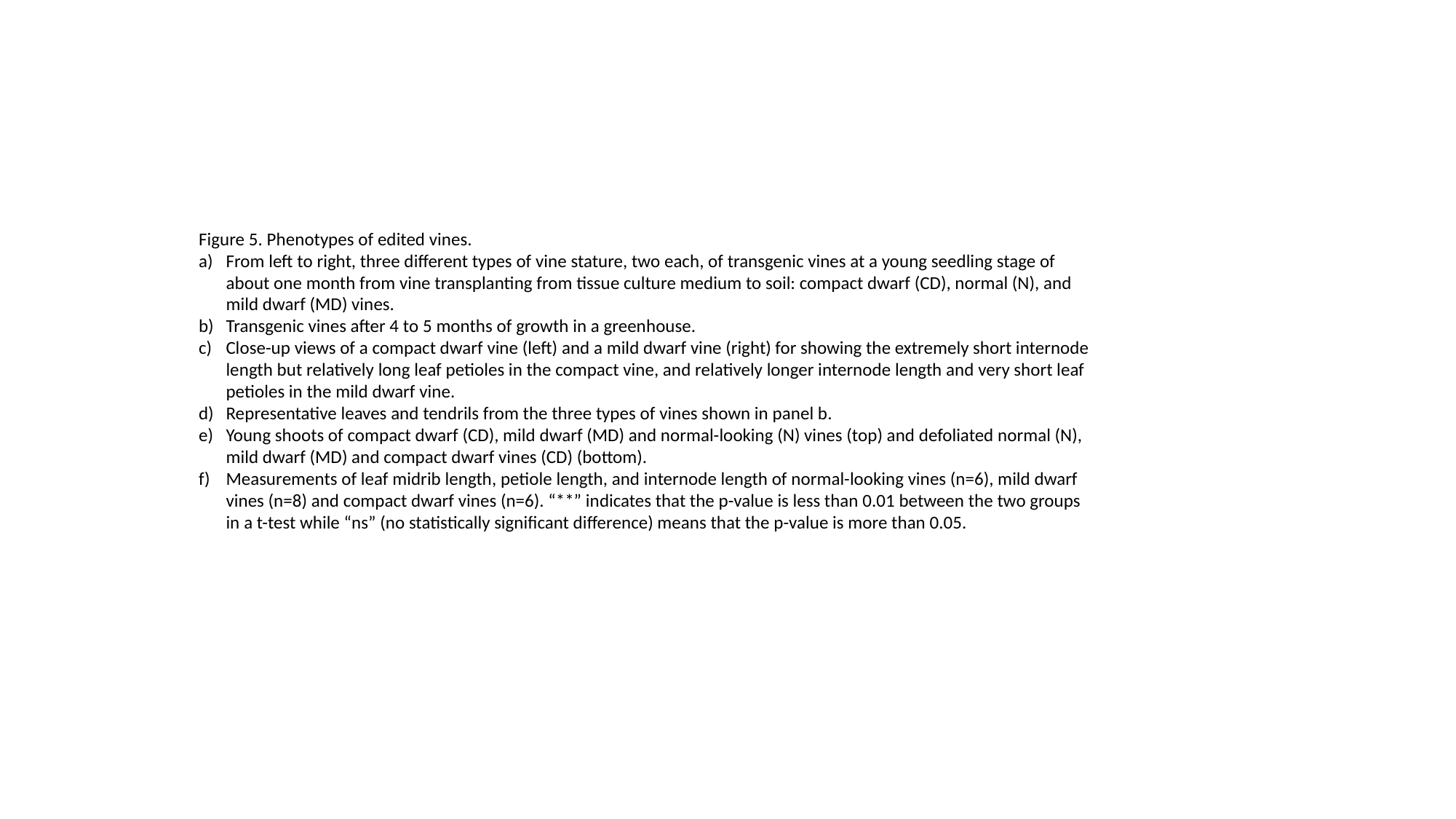

Figure 5. Phenotypes of edited vines.
From left to right, three different types of vine stature, two each, of transgenic vines at a young seedling stage of about one month from vine transplanting from tissue culture medium to soil: compact dwarf (CD), normal (N), and mild dwarf (MD) vines.
Transgenic vines after 4 to 5 months of growth in a greenhouse.
Close-up views of a compact dwarf vine (left) and a mild dwarf vine (right) for showing the extremely short internode length but relatively long leaf petioles in the compact vine, and relatively longer internode length and very short leaf petioles in the mild dwarf vine.
Representative leaves and tendrils from the three types of vines shown in panel b.
Young shoots of compact dwarf (CD), mild dwarf (MD) and normal-looking (N) vines (top) and defoliated normal (N), mild dwarf (MD) and compact dwarf vines (CD) (bottom).
Measurements of leaf midrib length, petiole length, and internode length of normal-looking vines (n=6), mild dwarf vines (n=8) and compact dwarf vines (n=6). “**” indicates that the p-value is less than 0.01 between the two groups in a t-test while “ns” (no statistically significant difference) means that the p-value is more than 0.05.

## Slide 7
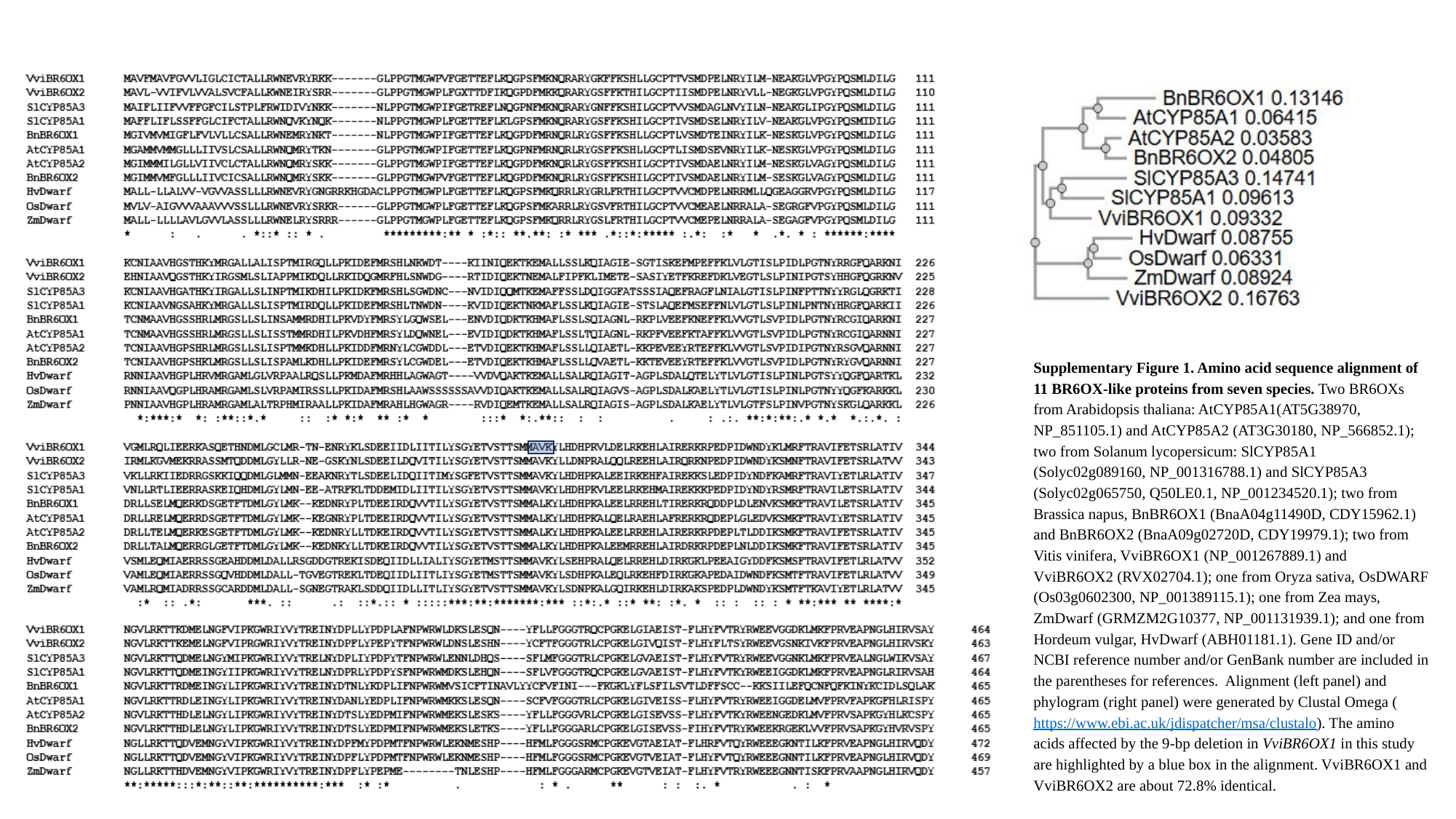

Supplementary Figure 1. Amino acid sequence alignment of 11 BR6OX-like proteins from seven species. Two BR6OXs from Arabidopsis thaliana: AtCYP85A1(AT5G38970, NP_851105.1) and AtCYP85A2 (AT3G30180, NP_566852.1); two from Solanum lycopersicum: SlCYP85A1 (Solyc02g089160, NP_001316788.1) and SlCYP85A3 (Solyc02g065750, Q50LE0.1, NP_001234520.1); two from Brassica napus, BnBR6OX1 (BnaA04g11490D, CDY15962.1) and BnBR6OX2 (BnaA09g02720D, CDY19979.1); two from Vitis vinifera, VviBR6OX1 (NP_001267889.1) and VviBR6OX2 (RVX02704.1); one from Oryza sativa, OsDWARF (Os03g0602300, NP_001389115.1); one from Zea mays, ZmDwarf (GRMZM2G10377, NP_001131939.1); and one from Hordeum vulgar, HvDwarf (ABH01181.1). Gene ID and/or NCBI reference number and/or GenBank number are included in the parentheses for references. Alignment (left panel) and phylogram (right panel) were generated by Clustal Omega (https://www.ebi.ac.uk/jdispatcher/msa/clustalo). The amino acids affected by the 9-bp deletion in VviBR6OX1 in this study are highlighted by a blue box in the alignment. VviBR6OX1 and VviBR6OX2 are about 72.8% identical.

## Slide 8
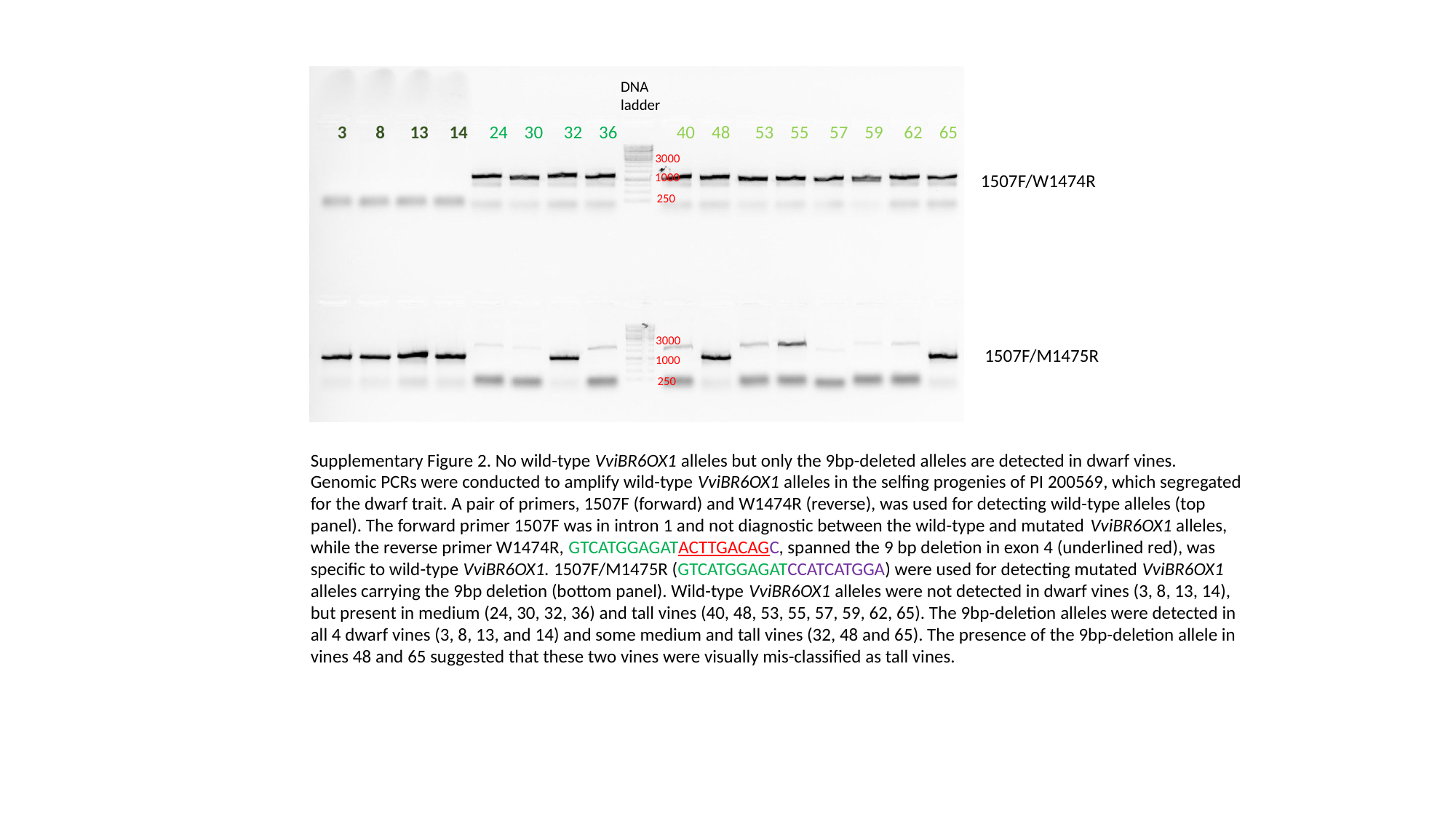

DNA
ladder
3 8 13 14 24 30 32 36 40 48 53 55 57 59 62 65
3000
1000
1507F/W1474R
250
3000
1507F/M1475R
1000
250
Supplementary Figure 2. No wild-type VviBR6OX1 alleles but only the 9bp-deleted alleles are detected in dwarf vines.
Genomic PCRs were conducted to amplify wild-type VviBR6OX1 alleles in the selfing progenies of PI 200569, which segregated for the dwarf trait. A pair of primers, 1507F (forward) and W1474R (reverse), was used for detecting wild-type alleles (top panel). The forward primer 1507F was in intron 1 and not diagnostic between the wild-type and mutated VviBR6OX1 alleles, while the reverse primer W1474R, GTCATGGAGATACTTGACAGC, spanned the 9 bp deletion in exon 4 (underlined red), was specific to wild-type VviBR6OX1. 1507F/M1475R (GTCATGGAGATCCATCATGGA) were used for detecting mutated VviBR6OX1 alleles carrying the 9bp deletion (bottom panel). Wild-type VviBR6OX1 alleles were not detected in dwarf vines (3, 8, 13, 14), but present in medium (24, 30, 32, 36) and tall vines (40, 48, 53, 55, 57, 59, 62, 65). The 9bp-deletion alleles were detected in all 4 dwarf vines (3, 8, 13, and 14) and some medium and tall vines (32, 48 and 65). The presence of the 9bp-deletion allele in vines 48 and 65 suggested that these two vines were visually mis-classified as tall vines.

## Slide 9
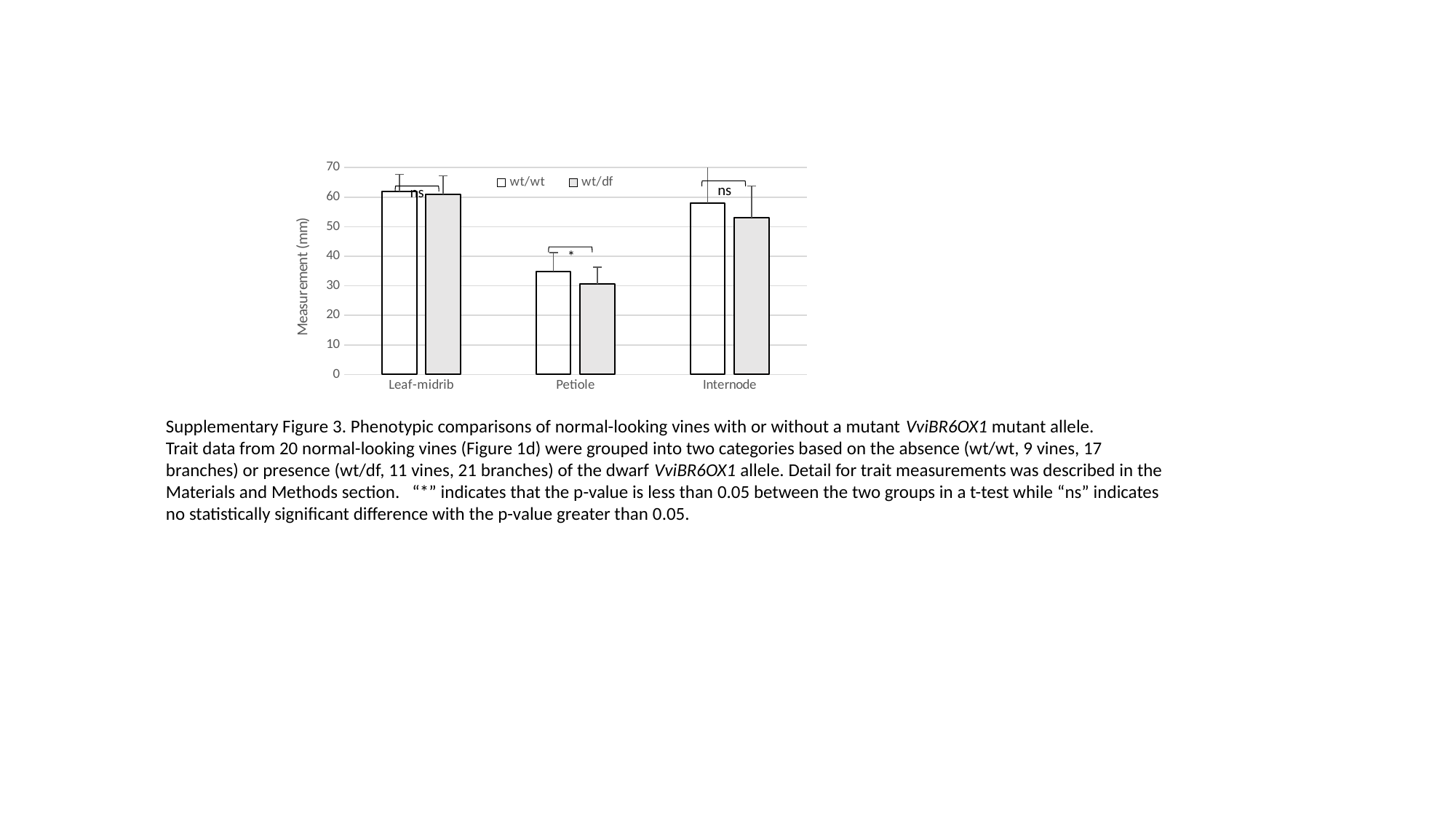

### Chart
| Category | wt/wt | wt/df |
|---|---|---|
| Leaf-midrib | 61.94677871148459 | 60.9501133786848 |
| Petiole | 34.86694677871148 | 30.59750566893424 |
| Internode | 57.99759903961585 | 53.13994169096211 |
ns
ns
*
Supplementary Figure 3. Phenotypic comparisons of normal-looking vines with or without a mutant VviBR6OX1 mutant allele.
Trait data from 20 normal-looking vines (Figure 1d) were grouped into two categories based on the absence (wt/wt, 9 vines, 17 branches) or presence (wt/df, 11 vines, 21 branches) of the dwarf VviBR6OX1 allele. Detail for trait measurements was described in the Materials and Methods section. “*” indicates that the p-value is less than 0.05 between the two groups in a t-test while “ns” indicates no statistically significant difference with the p-value greater than 0.05.

## Slide 10
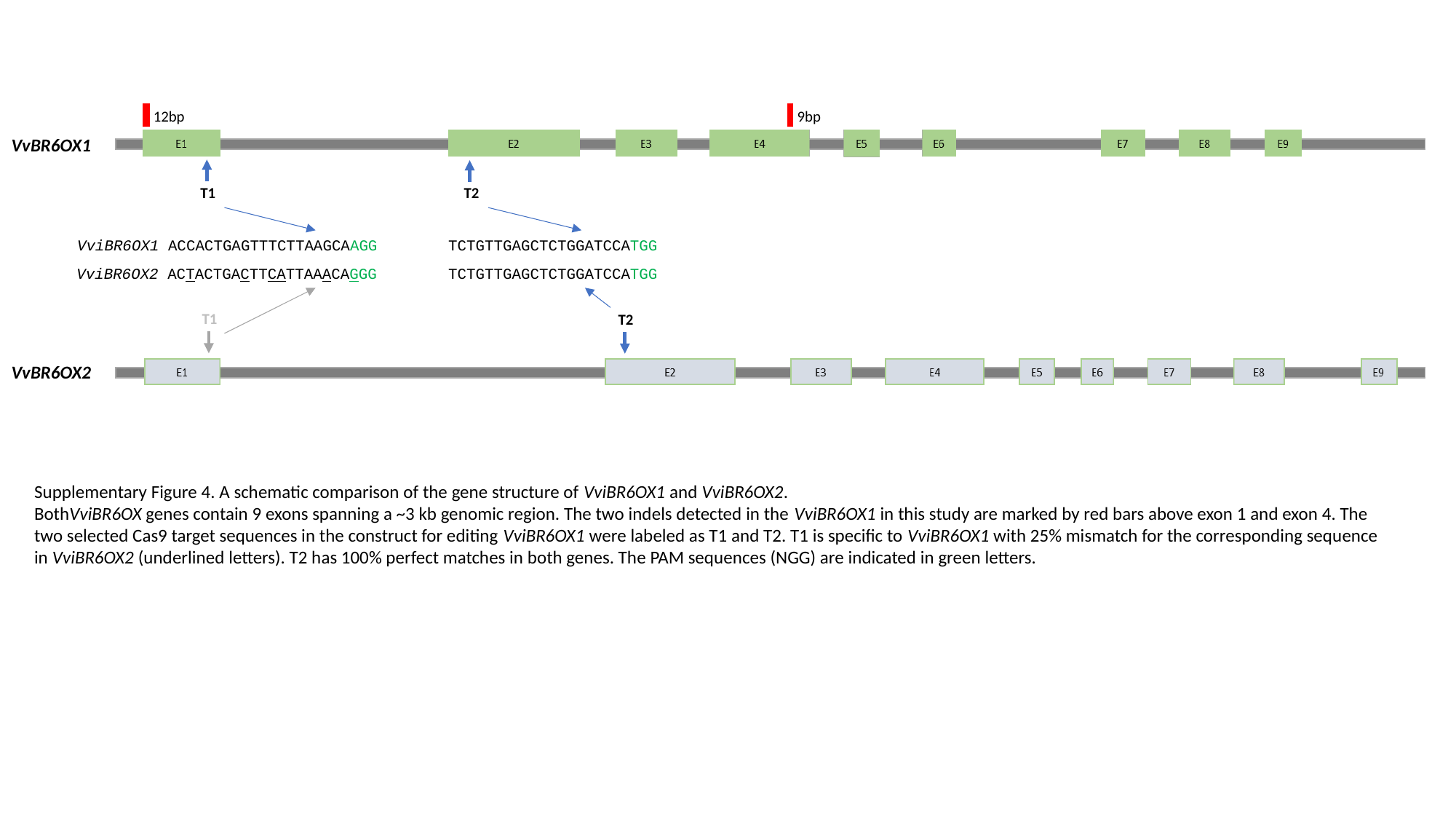

12bp
9bp
VvBR6OX1
T1
T2
VviBR6OX1 ACCACTGAGTTTCTTAAGCAAGG
TCTGTTGAGCTCTGGATCCATGG
VviBR6OX2 ACTACTGACTTCATTAAACAGGG
TCTGTTGAGCTCTGGATCCATGG
T1
T2
VvBR6OX2
Supplementary Figure 4. A schematic comparison of the gene structure of VviBR6OX1 and VviBR6OX2.
BothVviBR6OX genes contain 9 exons spanning a ~3 kb genomic region. The two indels detected in the VviBR6OX1 in this study are marked by red bars above exon 1 and exon 4. The two selected Cas9 target sequences in the construct for editing VviBR6OX1 were labeled as T1 and T2. T1 is specific to VviBR6OX1 with 25% mismatch for the corresponding sequence in VviBR6OX2 (underlined letters). T2 has 100% perfect matches in both genes. The PAM sequences (NGG) are indicated in green letters.

## Slide 11
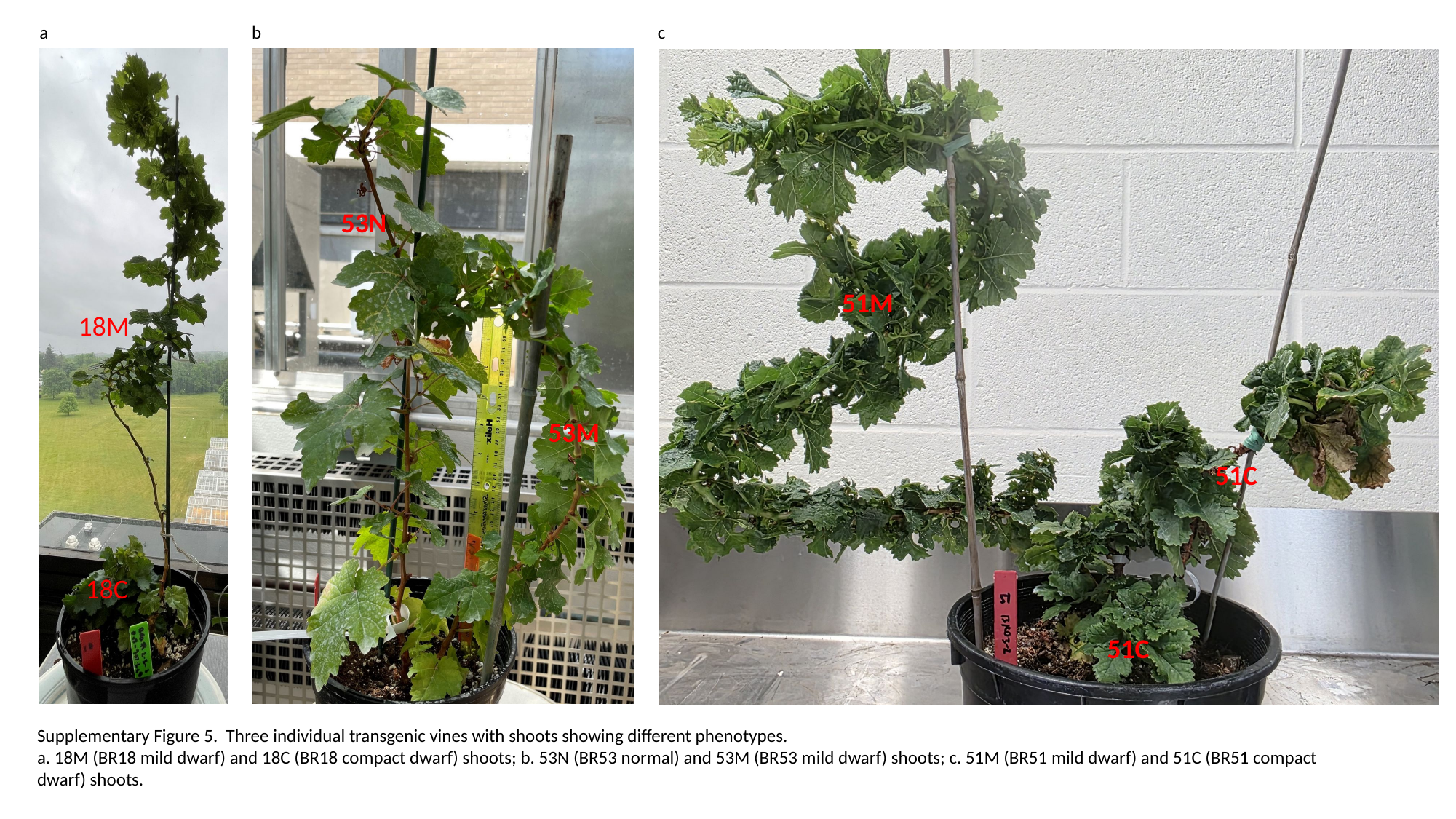

a
b
c
53N
Supplementary Figure 5. Two different vine phenotypes originated from the same transgenic vine.
a. Mild (18M) and compact (18C) dwarf
b. Normal (53N) and mild dwarf (53M)
51M
53M
18M
53M
51C
53N
18C
51C
Supplementary Figure 5. Three individual transgenic vines with shoots showing different phenotypes.
a. 18M (BR18 mild dwarf) and 18C (BR18 compact dwarf) shoots; b. 53N (BR53 normal) and 53M (BR53 mild dwarf) shoots; c. 51M (BR51 mild dwarf) and 51C (BR51 compact dwarf) shoots.

## Slide 12
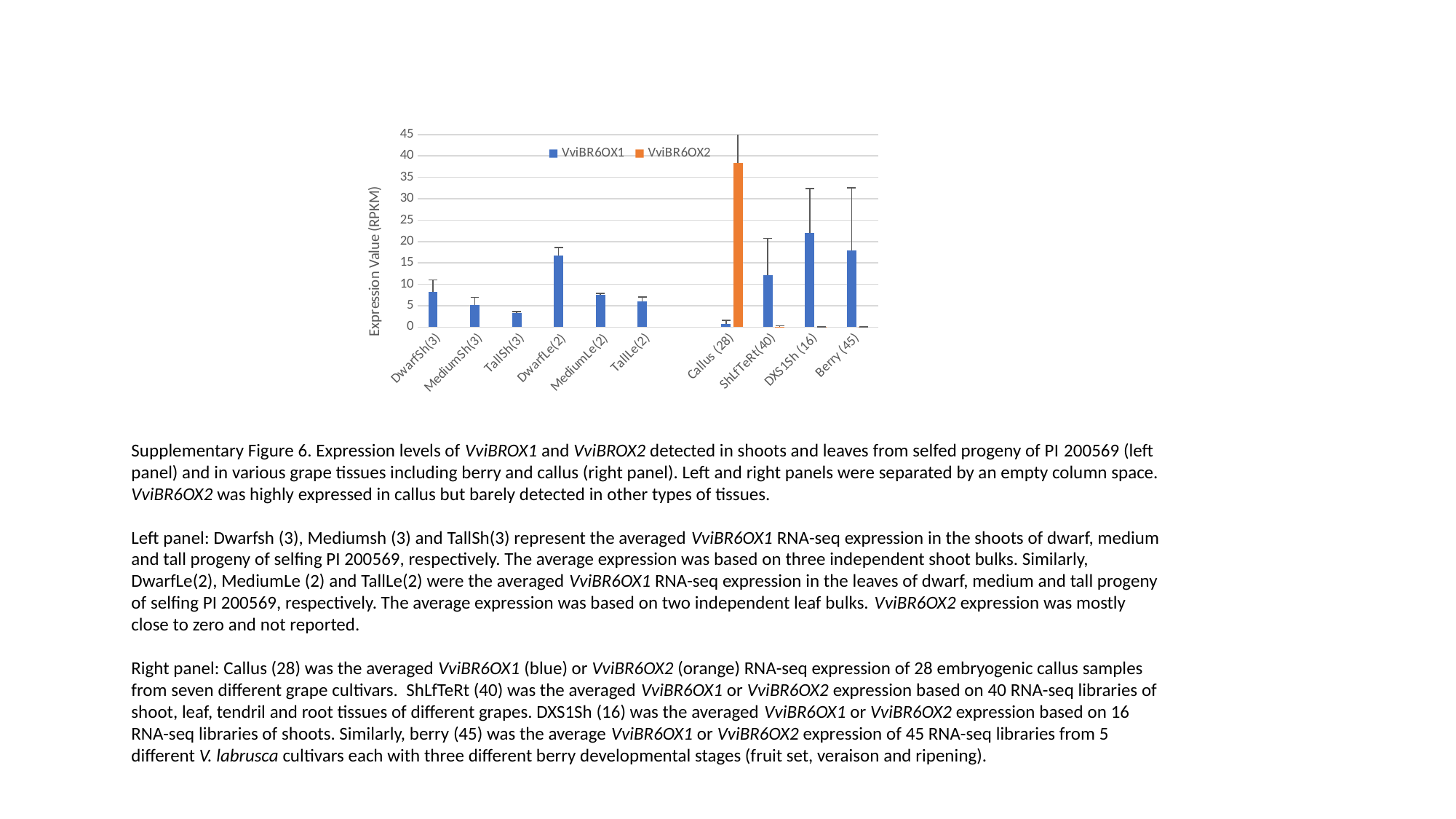

### Chart
| Category | VviBR6OX1 | VviBR6OX2 |
|---|---|---|
| DwarfSh(3) | 8.211094976045393 | None |
| MediumSh(3) | 5.14399472400184 | None |
| TallSh(3) | 3.326712527889846 | None |
| DwarfLe(2) | 16.794219966432287 | None |
| MediumLe(2) | 7.546127064394794 | None |
| TallLe(2) | 5.957535753790578 | None |
| | None | None |
| Callus (28) | 0.7250000000000002 | 38.3607142857143 |
| ShLfTeRt(40) | 12.131085384615384 | 0.10619207948717947 |
| DXS1Sh (16) | 21.985754000000004 | 0.0166766875 |
| Berry (45) | 17.93164597814663 | 0.017111584551126213 |Supplementary Figure 6. Expression levels of VviBROX1 and VviBROX2 detected in shoots and leaves from selfed progeny of PI 200569 (left panel) and in various grape tissues including berry and callus (right panel). Left and right panels were separated by an empty column space. VviBR6OX2 was highly expressed in callus but barely detected in other types of tissues.
Left panel: Dwarfsh (3), Mediumsh (3) and TallSh(3) represent the averaged VviBR6OX1 RNA-seq expression in the shoots of dwarf, medium and tall progeny of selfing PI 200569, respectively. The average expression was based on three independent shoot bulks. Similarly, DwarfLe(2), MediumLe (2) and TallLe(2) were the averaged VviBR6OX1 RNA-seq expression in the leaves of dwarf, medium and tall progeny of selfing PI 200569, respectively. The average expression was based on two independent leaf bulks. VviBR6OX2 expression was mostly close to zero and not reported.
Right panel: Callus (28) was the averaged VviBR6OX1 (blue) or VviBR6OX2 (orange) RNA-seq expression of 28 embryogenic callus samples from seven different grape cultivars. ShLfTeRt (40) was the averaged VviBR6OX1 or VviBR6OX2 expression based on 40 RNA-seq libraries of shoot, leaf, tendril and root tissues of different grapes. DXS1Sh (16) was the averaged VviBR6OX1 or VviBR6OX2 expression based on 16 RNA-seq libraries of shoots. Similarly, berry (45) was the average VviBR6OX1 or VviBR6OX2 expression of 45 RNA-seq libraries from 5 different V. labrusca cultivars each with three different berry developmental stages (fruit set, veraison and ripening).

## Slide 13
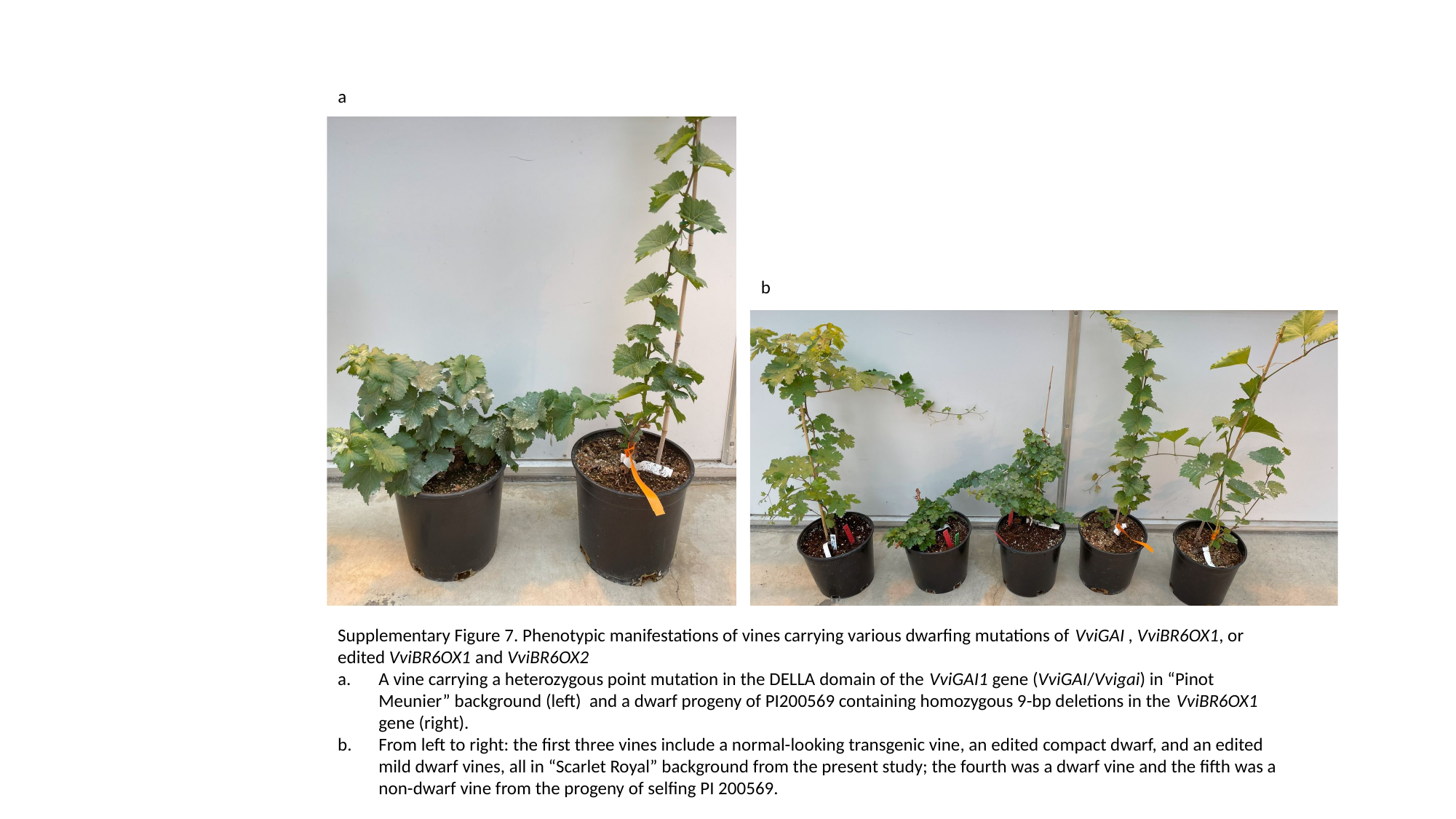

a
b
Supplementary Figure 7. Phenotypic manifestations of vines carrying various dwarfing mutations of VviGAI , VviBR6OX1, or edited VviBR6OX1 and VviBR6OX2
A vine carrying a heterozygous point mutation in the DELLA domain of the VviGAI1 gene (VviGAI/Vvigai) in “Pinot Meunier” background (left) and a dwarf progeny of PI200569 containing homozygous 9-bp deletions in the VviBR6OX1 gene (right).
From left to right: the first three vines include a normal-looking transgenic vine, an edited compact dwarf, and an edited mild dwarf vines, all in “Scarlet Royal” background from the present study; the fourth was a dwarf vine and the fifth was a non-dwarf vine from the progeny of selfing PI 200569.
